# Bacterial Symbionts in Lepidoptera: Their Diversity, Transmission, and Impact on the Host

**DOI:** 10.3389/fmicb.2018.00556

**Published:** 2018-03-27

**Authors:** Luis R. Paniagua Voirol, Enric Frago, Martin Kaltenpoth, Monika Hilker, Nina E. Fatouros

**Affiliations:** ^1^Institute of Biology, Freie Universität Berlin, Berlin, Germany; ^2^Centre de Coopération Internationale en Recherche Agronomique pour le Développement, Unité Mixte de Recherche Peuplements Végétaux et Bioagresseurs en Milieu Tropical, Saint-Pierre, La Réunion; ^3^Department for Evolutionary Ecology, Institute of Organismic and Molecular Evolution, Johannes Gutenberg University Mainz, Mainz, Germany; ^4^Biosystematics Group, Wageningen University and Research, Wageningen, Netherlands

**Keywords:** gut bacteria, endosymbionts, moth, butterfly, symbiosis, horizontal transfer, maternal transfer

## Abstract

The insect’s microbiota is well acknowledged as a “hidden” player influencing essential insect traits. The gut microbiome of butterflies and moths (Lepidoptera) has been shown to be highly variable between and within species, resulting in a controversy on the functional relevance of gut microbes in this insect order. Here, we aim to (i) review current knowledge on the composition of gut microbial communities across Lepidoptera and (ii) elucidate the drivers of the variability in the lepidopteran gut microbiome and provide an overview on (iii) routes of transfer and (iv) the putative functions of microbes in Lepidoptera. To find out whether Lepidopterans possess a core gut microbiome, we compared studies of the microbiome from 30 lepidopteran species. Gut bacteria of the Enterobacteriaceae, Bacillaceae, and Pseudomonadaceae families were the most widespread across species, with *Pseudomonas, Bacillus, Staphylococcus, Enterobacter*, and *Enterococcus* being the most common genera. Several studies indicate that habitat, food plant, and age of the host insect can greatly impact the gut microbiome, which contributes to digestion, detoxification, or defense against natural enemies. We mainly focus on the gut microbiome, but we also include some examples of intracellular endosymbionts. These symbionts are present across a broad range of insect taxa and are known to exert different effects on their host, mostly including nutrition and reproductive manipulation. Only two intracellular bacteria genera (*Wolbachia* and *Spiroplasma*) have been reported to colonize reproductive tissues of Lepidoptera, affecting their host’s reproduction. We explore routes of transmission of both gut microbiota and intracellular symbionts and have found that these microbes may be horizontally transmitted through the host plant, but also vertically via the egg stage. More detailed knowledge about the functions and plasticity of the microbiome in Lepidoptera may provide novel leads for the control of lepidopteran pest species.

## Introduction

Bacterial symbionts inhabiting insects can significantly impact the biology of their host (Douglas, 2015). These symbionts can be distinguished as intra- and extracellular based on whether they live within insect cells, or colonize the lumen or lining of insect cavities and body surface (Dillon and Dillon, 2004; Engel and Moran, 2013; Hansen and Moran, 2014). Symbionts are considered as primary or secondary, depending on whether they are needed by the host to survive or provide non-essential benefits (Douglas, 2015).

Obligatory symbionts are commonly harbored in specialized cells (bacteriocytes) and play important roles for nutrition in certain insect groups, particularly in phloem feeding taxa. For example, intracellular *Buchnera* bacteria associated with aphids provide essential amino acids and vitamins (Baumann et al., 1997; Hansen and Moran, 2014). The benefits provided by secondary symbionts, on the other hand, are often context-dependent. In aphids, for example, secondary symbionts can provide a range of ecological benefits including resistance to pathogens and parasitoids, and heat tolerance, but they can be costly under benign conditions (Oliver et al., 2010). Some *Wolbachia* strains living intracellularly can manipulate host reproduction to favor their own spread in the population (Werren et al., 2008; Zug and Hammerstein, 2015), while others can be facultative (Teixeira et al., 2008) or even become obligatory in certain species (Hosokawa et al., 2010).

The composition and robustness of gut bacterial communities varies extensively across the animal kingdom ranging from more than 1,000 phylotypes in humans (Lozupone et al., 2012), over several hundreds in termites (Hongoh et al., 2005; Brune and Dietrich, 2015), and a few tens in lepidopterans (Broderick et al., 2004; Robinson et al., 2010; Pinto-Tomás et al., 2011), to an almost complete absence of bacteria in aphid guts (Douglas, 1988; Grenier et al., 2006). In insects, the best studied and most diverse gut bacterial communities are those belonging to groups feeding on wood, decaying matter, or detritus such as termites, cockroaches, crickets, and some beetles (Dillon and Dillon, 2004; Engel and Moran, 2013). Gut bacterial communities often deliver metabolic benefits to their hosts by the provision of digestive enzymes and production of vitamins, thus improving nutrient uptake on deficient diets (Dillon and Dillon, 2004; Anand et al., 2010; Engel and Moran, 2013; Salem et al., 2015). Furthermore, they can provide protection against pathogens (Dillon and Dillon, 2004) and support detoxification of pesticides or harmful plant secondary metabolites (Kikuchi et al., 2012; van den Bosch and Welte, 2017; Xia et al., 2017).

Lepidoptera comprise the second most diverse insect order with some of the most devastating agricultural pests worldwide (Sree and Varma, 2015). Yet, clear evidence for bacterial associates playing a fundamental role in lepidopteran biology is scarce. The functional role of the gut microbiome of Lepidoptera has been challenged by a recent study reporting that caterpillars harbor no or only few resident bacteria when compared to other insect orders (Hammer et al., 2017). The authors of this study argue that this is probably due to caterpillars being rough environments for bacterial colonization, because they possess an unusually alkaline gut with a rapid food passage of approximately two hours. In addition lepidopterans undergo a holometabolous metamorphosis which entirely re-shapes their body structures (Anand et al., 2010). In spite of this harsh environment for the gut microbiota, several studies have shown that bacteria do affect essential physiological functions in Lepidoptera, i.e., facilitation of nutrient acquisition and digestion (Pinto-Tomás et al., 2007; Indiragandhi et al., 2008; Xia et al., 2017), overcoming plant anti-herbivore defenses (Visotto et al., 2009; Xia et al., 2017), or strengthening of immune responses for protection against pathogens (Shao et al., 2017).

Out of the 157,424 recognized lepidopteran species (Mitter et al., 2017), <0.1% have been screened for bacterial associates, which reveals that our knowledge on bacterial associates in Lepidoptera is still limited. Many of these studies focused on specific endosymbionts known to be widespread in arthropods, such as *Wolbachia* and *Spiroplasma*. Many other studies are mostly descriptive and focused on larvae, while only a few have addressed the potential impact on their host traits. These studies screened bacteria from specimens of several of the ∼43 lepidopteran superfamilies (Mitter et al., 2017), i.e., Hepialoidea, Yponomeutoidea, Tortricoidea, Cossoidea, Papilionidea, Gelechioidea, Pyralioidea, Depranoidea, Noctuoidea, Geometroidea, and Bombycoidea (Supplementary Table 1). Knowledge on whether certain bacteria taxa are persistent across the Lepidoptera order is limited, as well as information on how Lepidoptera transfer symbiotic bacteria among individuals of a population and between generations.

Our review aims to find hints for answering these questions and points to future studies by screening the current literature on microbial associates in Lepidoptera. We surveyed the literature to assess which bacterial taxa were detected in independent studies comprising 30 different lepidopteran species, and asked which ones are ubiquitous in these taxa. We further considered potential drivers explaining the variability found in the composition of the lepidopteran gut microbiome. These drivers include ecological, morphological, and developmental traits of Lepidoptera. These features significantly impact the way by which bacterial symbionts are transmitted between individuals and through generations. Understanding the role of symbiotic bacteria in such an economically important insect order may provide novel leads for improving current integrated pest management techniques. This knowledge is also important from a fundamental perspective to understand the role that symbiotic bacteria play in helping lepidopteran larvae to cope with challenges such as diet deficiencies, host plant switches, and natural enemy attacks.

### Composition of the Gut Microbiota in Lepidoptera

**FIGURE 1:**
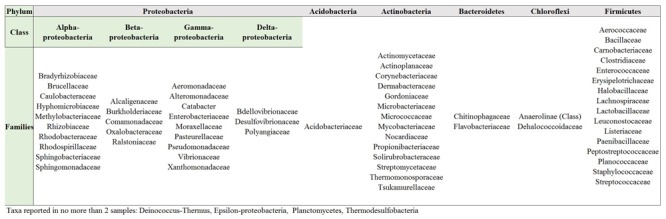
Bacteria families in Lepidoptera. Most families (ca. 42%) belong to the Proteobacteria phylum. Compare Supplementary Table 1 for information on the Lepidopteran taxa considered and the references.

**FIGURE 2:**
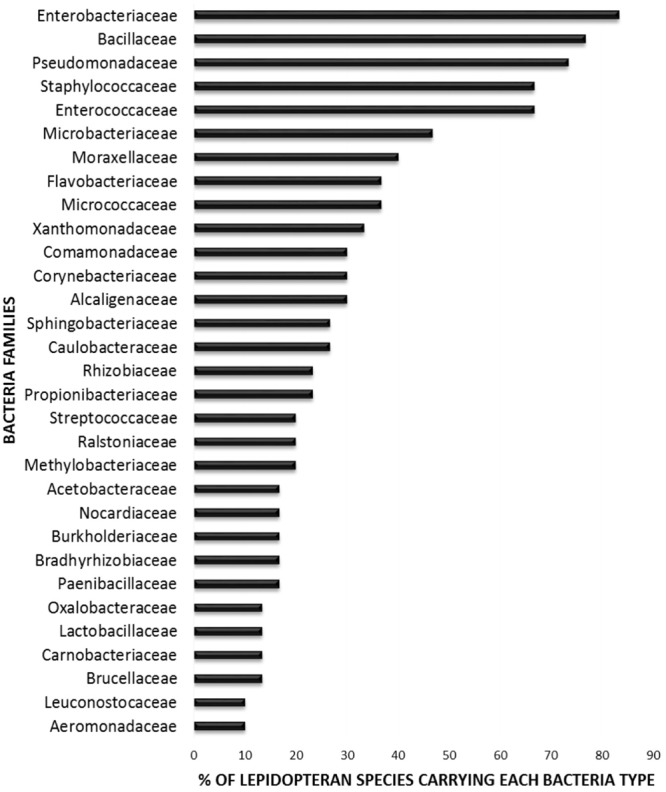
Percentage of lepidopteran species hosting the most common bacteria families. The graph comprises independent studies on 30 Lepidoptera species, considering only their gut communities. Families present in less than three Lepidoptera species are not depicted. For detailed information on each study see Supplementary Table 1.

**FIGURE 3:**
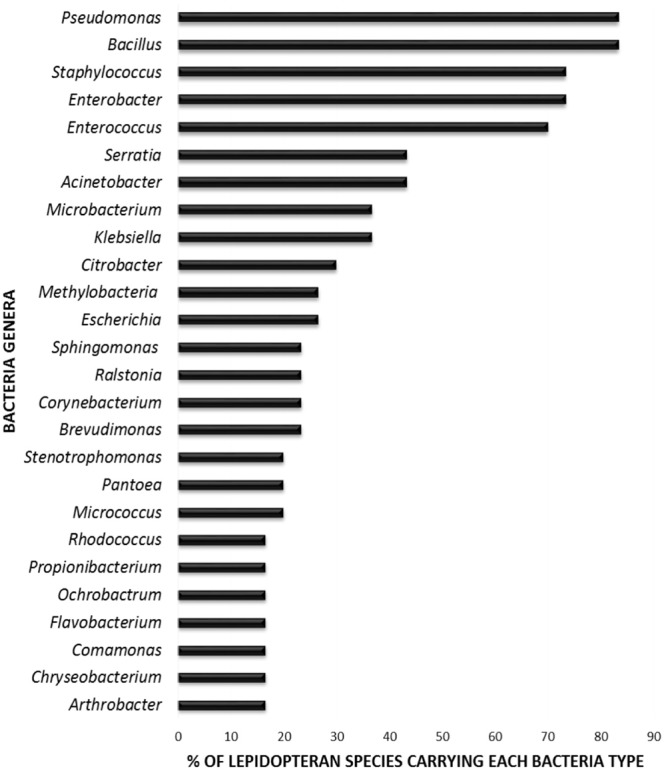
Percentage of lepidopteran species hosting the most commonly reported bacterial genera. The graph comprises independent studies on 30 Lepidoptera species. Those gut members that were identified only at a higher taxonomic level (i.e., family level) are not considered. Genera present in less than three Lepidoptera species are not depicted. For detailed information on each study see Supplementary Table 1.

In order to elucidate whether some bacterial taxa are ubiquitous in the gut of Lepidoptera, we screened independent studies comprising 30 different lepidopteran species. Despite the differences in the methodology used in the different studies, such as differences in the life stage, insect diet, and screening technique (culture-based, cloning/sequencing, or high-throughput amplicon) (see Supplementary Table 1), our survey based on presence/absence shows that certain bacteria taxa are widespread across lepidopterans. Most of the detected gut bacterial families belong to the Proteobacteria phylum (42%) (**Figure 1**). Within this group, those families belonging to the α- and γ-Proteobacteria classes are the most common (72%) (**Figure 1**). Bacteria belonging to the Enterobacteriaceae, Bacillaceae, Pseudomonadaceae, Staphylococcaceae, and Enterococcaceae families are present in >60% of the screened lepidopteran species (**Figure 2**). At the genus level, the most widespread bacteria belong to *Pseudomonas, Bacillus, Staphylococcus, Enterobacter*, and *Enterococcus*, each being present in >70% of the studied lepidopteran species (**Figure 3**). Persistence of some gut bacterial species occurs regardless of the diet the insects fed upon, indicating the presence of a core community (Broderick et al., 2004; Xiang et al., 2006; Robinson et al., 2010; Pinto-Tomás et al., 2011; Xia et al., 2013). Despite this, various studies also indicate that the gut microbiome shows great variability across lepidopteran species and even within a species, a question that will be discussed in detail in the following section.

### Drivers of Variability in Bacterial Gut Communities

The high variability of the lepidopteran gut microbiome could be promoted by different drivers, which may act alone or in concert and include the environment, insect diet, insect developmental stage, and gut physiology. Firstly, the environment where insects live affects the composition of the insects’ microbiome. Insects reared in the laboratory or collected in the field show different microbial communities even if they feed on the same host plant (e.g., Staudacher et al., 2016). The habitat may thus significantly affect the bacteria associated with lepidopteran species (Staudacher et al., 2016). Secondly, diet can have a major influence on bacterial community variability. Recent studies could not (Staudacher et al., 2016) or hardly (Hammer et al., 2017) detect any resident, host insect-specific, and food-independent bacteria in Lepidoptera. The bacterial community can therefore be expected to differ significantly between oligophagous and polyphagous species, or between herbivorous and carnivorous species. A comparative study on microbial communities associated with herbivorous and carnivorous Lycaenidae larvae, however, did not find consistent patterns in community profiles that could relate them to the diet of the insect (Whitaker et al., 2016). By contrast, an assessment on the influence of diet and host taxonomy on gut bacterial communities across several insect orders found that, depending on the insect taxon, either factor was significant (Colman et al., 2012). Insects feeding on decaying matter presented the richest communities, while bees and wasps had the lowest. While host taxonomy was an important driver of bacterial communities in hymenopterans and termites, diet was important in insects feeding on lignocellulose-derived components. Non-conclusive patterns of clustering among lepidopterans were found, based on a rather small number of species studied (Colman et al., 2012).

In addition to diet and environment, the developmental stage can influence the host’s gut microbiota. Concordantly, instar-specific bacterial communities were detected in larvae of the moth *Spodoptera littoralis* (Chen et al., 2016). As in all other holometabolous insect orders, metamorphosis in Lepidoptera entails major morphological rearrangements and is usually accompanied by a change in diet, which can have a strong impact on gut microbiota composition. While almost all lepidopteran species feed upon plant tissue during their larval stage (with a few notable carnivorous and fungivorous exceptions), the adult stage of most species feeds on nectar (Strong et al., 1984). With the proviso that gut communities depend on the diet, it is not surprising that bacterial communities differ considerably between larvae and adults of the same species (Staudacher et al., 2016; Xia et al., 2017). Nevertheless, certain taxa may persist throughout the entire insect life cycle as shown for bacteria species belonging to the families Acetobacteraceae, Moraxellaceae, Enterobacteriaceae, Enterococcaceae, Streptococcaceae, and unclassified Bacteroidetes, which dominate the gut of the larval, pupal, and adult stages of the red postman (*Heliconius erato*) (Hammer et al., 2014). Some bacteria like *Enterococcus mundtii* may even survive and propagate in the digestive tract of *S. littoralis* across its life cycle, and persist up to two consecutive generations (Teh et al., 2016). Such persistence of some bacterial symbionts across the entire development is also found in other holometabolous insects that inhabit different ecological niches during the larval and adult stage, like the emerald ash borer beetle *Agrilus planipennis* (Vasanthakumar et al., 2008), the fruitfly *Ceratitis capitata* (Lauzon et al., 2009), or the scarabaeid beetle *Melolontha hippocastani* (Arias-Cordero et al., 2012). Thus, while some core bacteria persist in holometabolous insects including Lepidoptera, a considerable change in the bacterial community composition from larvae to adults is common. This is probably due to the physiological changes occurring during metamorphosis, and also due to the change in diet from larvae to adults, which is particularly dramatic in Lepidoptera.

Persistence of bacteria in the gut of lepidopteran larvae is further impeded by the lack of intricate pouches-like gut structures that are known to harbor bacterial symbionts in other insect taxa (Alonso-Pernas et al., 2017; Sudakaran et al., 2017). A complex anatomy of the gut with a high number of pouches (diverticula, caeca) might favor the establishment of a robust bacterial community, as seen in other non-lepidopteran insects with extremely rich bacterial gut communities. For example, termites with their complex gut structures harbor highly robust gut communities that vary across the gut compartments (Dillon and Dillon, 2004; Anand et al., 2010; Barbehenn and Peter Constabel, 2011; Engel and Moran, 2013).

### Main Transmission Routes of Gut Bacteria: Vertically or Horizontally?

How Lepidoptera gain and retain gut bacterial members is a largely unresolved question. It is still unclear to which extent gut symbiotic bacteria are transmitted either (a) vertically from one generation to the next or (b) horizontally between individuals directly via contact among individuals or indirectly via uptake from the diet (Hammer et al., 2017; Hurst, 2017). Detection of core bacterial associates in the gut suggests a potential vertical transmission or a consistent horizontal acquisition of gut symbionts in some species, while environmental uptake of transient associates appears to be likely in others (Staudacher et al., 2016).

**FIGURE 4:**
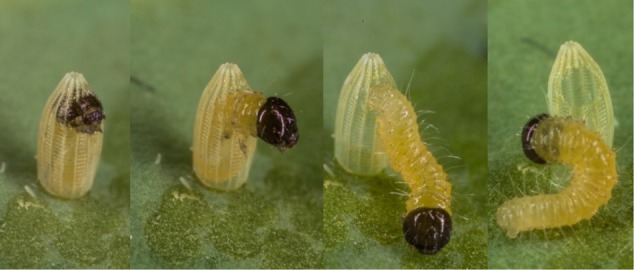
Caterpillar of large cabbage white butterfly *Pieris brassicae* hatching from egg and feeding on egg shell. Many insect larvae take up symbionts maternally transferred via the egg, e.g., smeared over the egg surface following oviposition (see Salem et al., 2015). Evidence for vertical transmission of extracellular symbionts in Lepidoptera is still scarce. Photo credits: NEF.

Since gut bacteria live extracellularly, they are probably not transmitted inside insect oocytes, although translocation of gut bacteria to the oocytes was reported in *Galleria mellonella* (Freitak et al., 2014). However, these authors did not test whether such bacteria remain viable in the following generation. In some insect orders like Heteroptera, extracellular bacteria are added to the egg surface by the females in secretions or feces, which are later acquired by the hatching nymphs (Salem et al., 2015). Transmission of extracellular symbionts via the egg stage requires that bacteria remain alive before colonizing the newly emerged larvae. This would be possible if symbionts are deposited in an inactive stage, or if active bacteria are nourished through the egg shell or egg-associated secretions. In either case, transmission via the egg stage requires that larvae take up these bacteria when hatching. Since neonate lepidopteran larvae bite through their egg shell while hatching and often fully ingest it after hatching (**Figure 4**), infection of neonate larvae with bacteria on the outer egg surface is possible.

The presence of insect gut bacteria associated with the eggs has been shown for some lepidopterans (Brinkmann et al., 2008; Pinto-Tomás et al., 2011; Tang et al., 2012; Mason and Raffa, 2014). Yet, only for the tobacco hornworm (*Manduca sexta*), bacterial (*Enterococcus*) metabolic activity has been confirmed during the egg stage (Brinkmann et al., 2008). The fact that these bacteria are metabolically active when associated with *M. sexta* eggs suggests a high metabolic adaptability which allows them to survive both in association with the eggs and later in the gut of larvae (Brinkmann et al., 2008). However, metabolic activity is not strictly necessary for successful vertical transmission, since some inactive egg-associated bacteria may also re-activate their metabolism once reaching the gut of the neonate. Active bacterial growth during the egg stage may increase their chances to colonize not only the hatching neonates but also their environment (e.g., soil or host plant), which could ultimately promote horizontal transmission. It would be relevant to quantify bacterial growth during the egg incubation phase to elucidate bacterial proliferation since the beginning of insect development.

Intra- and extracellular symbionts in herbivorous insects are known to be also acquired through their host plant (Caspi-Fluger et al., 2012; Li et al., 2016; Chrostek et al., 2017; Flórez et al., 2017), which is another example that indicates an enormous adaptability of the bacteria to various habitats. Despite these evidences, bacteria present on the host plant may also reflect the bacteria community present in the habitat, and studies of bacterial associates in Lepidoptera should always include the appropriate controls to disentangle whether the detected bacteria originate from the environment or are specific for the insect.

As outlined above, many gut bacteria found in Lepidoptera are ubiquitous. For example, *Enterococcus* has been detected in many lepidopteran species and other insect orders (Hunt and Charnley, 1981; Cox and Gilmore, 2007; Lehman et al., 2009; Tang et al., 2012; Schmid et al., 2014). A comparative study of *Heliothis virescens* butterflies obtained from either the laboratory or the field revealed that *Enterococcus* was dominant in individuals of the laboratory strain, but absent in those from the field, suggesting that the presence of this bacterium is a laboratory artifact in this species (Staudacher et al., 2016). Due to the high prevalence of *Enterococcus* in different habitats and conditions (Fisher and Phillips, 2009), these bacteria might be simply acquired from the laboratory environment, and their presence on the eggs might not represent a natural condition.

With such scarce evidence of insect-specific gut bacteria being present on eggs, it remains speculative to consider eggs as a vehicle for vertical symbiont transmission in Lepidoptera. More studies are therefore needed to determine to what extent the bacterial members persist across generations to pinpoint those bacterial members that potentially share a long co-evolutionary history with their lepidopteran host. Bacterial symbionts that share a longer history with their host and that are vertically transmitted are most likely playing fundamental roles in the host biology. However, symbionts acquired *de novo* from the environment every generation could also be functionally important as was found in the bean bug (*Riptortus pedestris*), which developed insecticide resistance after acquiring insecticide-degrading bacteria (*Burkholderia*) from the soil (Kikuchi et al., 2012).

### Gut Bacteria and Their Impact on Digestion and Nutrient Acquisition in Lepidoptera

Dietary nitrogen is particularly limiting in the diet of phloem-feeding insects which have evolved associations with amino acid- and vitamin-supplementing intracellular symbionts. Considering the high carbon-to-nitrogen ratio in leaves, it is likely that chewing insects also need to cope with limited dietary nitrogen (Waldbauer and Friedman, 1991; Schoonhoven et al., 2005). Symbiotic bacteria may be beneficial by fixing nitrogen and converting it into physiologically relevant nitrogen-containing compounds (Nardi et al., 2002). Rhizobacteria fixing molecular dinitrogen into ammonium are well known to be associated with plant roots (Vance, 2001; Werner et al., 2014). Some insects like termites harbor bacteria which also have the ability to fix dinitrogen into ammonia which is then assimilated by gut endosymbionts that biosynthesize vitamins and amino acids needed for insect development (van Borm et al., 2002; Frohlich et al., 2007; Kneip et al., 2007; Brune, 2014; Brune and Dietrich, 2015). A study on the moth *Plutella xylostella* demonstrated that the same may occur in Lepidoptera, as many bacteria isolated from the gut were found to be able to fix nitrogen *in vitro* (Indiragandhi et al., 2008).

Nitrogen present in hairs and feathers in the form of keratin, a cysteine-rich polypeptide, is the nutritional, nitrogen-rich resource of a few lepidopteran species like the clothes’ moth *Tineola bisselliella.* This species can break the cysteine-bonds in keratin thus making the polypeptide more accessible to proteases for digestion. Since keratinases are only known from bacteria and fungi (Hughes and Vogler, 2006), it is possible that a microbial partner is involved in this digestion. Despite this, the mechanism by which *T. bisselliella* uses keratin is yet to be clarified, firstly because Crewther and Mcquade (1955) did not find any culturable bacterial colonies in the gut that could play this role, and secondly because the complete microbiome of this species has not been characterized. In addition, genes that resemble bacterial sequences encoding keratinolytic enzymes were not found in the genome of this moth in a later study (Hughes and Vogler, 2006).

Plant cell wall degrading enzymes (PCWDEs) include cellulases, hemicellulases, and pectinases that originate from insects and are responsible for the uptake of carbohydrates via the break-up of plant cell walls. The genes encoding for some of these enzymes are found in crickets (Orthoptera), stick insects (Phasmatodea), cockroaches and termites (Dictyoptera), lice (Phthiraptera), aphids (Homoptera), beetles (Coleoptera), and bees and wasps (Hymenoptera) (Watanabe and Tokuda, 2010). Phylogenetic analyses revealed that in many cases, these genes encoding PCWDEs were originally horizontally acquired from microorganisms and integrated into the host genome (Pauchet and Heckel, 2013; Kirsch et al., 2014; Shelomi et al., 2016a,b). However, the tortoise leaf beetle *Cassida rubiginosa* still maintains a bacterial symbiont that degrades pectin (Salem et al., 2017), a ubiquitous recalcitrant component of all plant tissues. So far, evidence for PCWDEs-encoding genes or symbionts in Lepidoptera is scarce (International Silkworm Genome Consortium, 2008), although Cong et al. (2015) found evidence for hypothetical cellulose-encoding genes in the hesperiid butterfly *Lerema accius*. These genes, however, were not detected in a close hesperiid relative, *Achalarus lyciades* (Shen et al., 2017). The presence of cellulase-encoding genes in ancestral insect taxa and its lack in most of the studied Lepidoptera may suggest that lepidopteran species lost these genes and may rely on symbionts for cellulose digestion. One of these examples may include *P. xylostella* the gut microbiome of which has thousands of genes from six families that encode carbohydrate-active enzymes, including cellulases (Xia et al., 2017).

Numerous other enzymes may be provided by bacteria inhabiting the lepidopteran gut. For example, bacteria present in caterpillars and pupae of the saturniid moths *Automeris zugana* and *Rothschildia lebeau* provide gelatinase, caseinase, lipase, esterase, and chitinase activity (Pinto-Tomás et al., 2007). These bacterial enzymatic activities might become especially important for efficient food digestion by the host insect during periods of food shortage or after host plant shifts (Genta et al., 2006; Anand et al., 2010). Since these bacteria were detected in pupae, it is also possible that they assist during metamorphosis, which is a very active event in terms of metabolic activity. For example, symbionts may provide chitinases for cuticle digestion, or aromatic amino acids for the synthesis of the new cuticle as suggested by Vigneron et al. (2014).

### Protection Against Entomopathogens by Lepidopteran Gut Bacteria

In addition to helping with nutrient acquisition, resident gut bacteria can provide protection against pathogens (Dillon and Dillon, 2004; Florez et al., 2015). One way to achieve this is by outcompeting pathogens, the so-called colonization resistance (Dillon and Dillon, 2004), as found in *Homona magnanima* whose caterpillars are more susceptible to *Bacillus thuringiensis* bacteria when they are reared aseptically than when they are not (Takatsuka and Kunimi, 2000). *Enterococcus faecalis* found in the gypsy moth is known to acidify its local environment so that it can colonize alkaline niches. This probably protects the gut against pathogenic toxins that are activated in alkaline conditions, such as those from *B. thuringiensis* (Broderick et al., 2004). The gut bacterial communities in some insects produce bactericidal substances that selectively target foreign bacteria, but they do not affect autochthonous ones (Dillon and Dillon, 2004). *E. mundtii*, for instance, is a highly abundant bacterium in the gut of *S. littoralis*, which produces an antimicrobial compound against Gram-positive pathogens like *Listeria*, but not against resident gut bacteria (Shao et al., 2017).

Some common gut bacterial inhabitants may be detrimental or beneficial depending on the community composition of the gut. One of such bacteria may be *Serratia* spp., a genus of bacteria commonly reported in lepidopterans and known to be pathogenic in many animals (Chadwick et al., 1990; Ishii et al., 2014). It would be relevant to evaluate if these bacteria provide benefits under certain conditions and whether they switch to a virulent phase when the structure of the bacterial community is altered. If the virulence of *Serratia* spp. depends on the composition of the entire insect microbial community, this would indicate a community-wide role in preventing pathogenic outbursts (Broderick et al., 2004). The role of gut bacteria in protecting lepidopterans against pathogens should be thus studied in a community context. For instance, while aseptic rearing in some cases results in less susceptibility to *B. thuringiensis* (Takatsuka and Kunimi, 2000), for *Lymantria dispar* it was found that the midgut community is actually required for the activity of *B. thuringiensis* toxin (Broderick et al., 2006).

Several studies have shown that exposure of insects to certain pathogens or parasites can boost the immune system of their offspring, an effect known as transgenerational immune priming (Sadd and Schmid-Hempel, 2007; Roth et al., 2010; Moreau et al., 2012; Trauer-Kizilelma and Hilker, 2015). For example, oral uptake of pathogenic bacteria by female caterpillars of the greater wax moth *G. mellonella* resulted in the increased expression of immunity-relevant genes in the caterpillars but also in the eggs laid by adult females developing from these caterpillars (Freitak et al., 2014). Transgenerational immune priming by orally ingested bacteria has also been observed in the moth *Trichoplusia ni* (Freitak et al., 2009). It has been proposed that such priming in Lepidoptera occurs by means of maternal transfer of bacteria or bacteria-associated compounds to the developing eggs (Freitak et al., 2014), which reinforces the idea that gut microbes can play an important role in insect defense against natural enemies.

### Lepidopteran Gut Bacteria That Counteract Anti-herbivore Plant Defenses

Plants defend themselves by a plethora of physical and chemical weapons against insect herbivory (Kessler and Baldwin, 2002; Despres et al., 2007; Mithofer and Boland, 2012; Hilker and Fatouros, 2016). Numerous plant secondary compounds with deterrent, anti-digestive, or toxic effects on insect herbivores are known. Some microbial symbionts can play a fundamental role in promoting the pest status of certain insect species by detoxifying plant allelochemicals (van den Bosch and Welte, 2017). Bacterial symbionts of insects can also influence insect–plant interactions to a greater extent than previously thought by helping their hosts manipulate the induction of plant defenses (Frago et al., 2012, 2017; Douglas, 2015).

A general strategy to detoxify plant lipophilic toxins is to convert them into water-soluble compounds which can easily be excreted. To achieve this, lipophilic toxins are usually functionalized (e.g., oxidized) and then conjugated with a highly polar compound, like glutathione. Bacteria in the gut of the diamondback moth (*P. xylostella*) are known to provide glutathione-*S*-transferase, an enzyme involved in this conjugation process (Xia et al., 2017).

Phenolic compounds, almost ubiquitously present in plants, can impair digestion of proteins through interactions with plant proteins and insect digestive enzymes. These compounds promote the production of reactive oxygen species (ROS) when they are oxidized into quinones, especially at alkaline pH values as those present in the lepidopteran gut (Appel, 1993; Salminen and Karonen, 2011). High concentrations of ROS, which are highly aggressive compounds interacting with almost all cellular components, may significantly damage cells (e.g., Bi and Felton, 1995). Bacteria in the lepidopteran gut, e.g., *Enterobacter* spp., can provide ROS-detoxifying enzymes like superoxide dismutase or catalase (Xia et al., 2017).

Terpenes, another class of secondary metabolites that are widespread in the plant kingdom, are toxic to many insects and bacteria because they may disturb chemiosmosis when lipophilic non-oxidized terpenes promote interactions with cell membranes (Gershenzon and Dudareva, 2007). For conifer-feeding bark beetles and weevils, gut symbionts have been implicated in the detoxification of terpenes that allow the insects to subsist on the terpene-rich diets (Adams et al., 2013; Wang et al., 2013; Berasategui et al., 2017). The gypsy moth can tolerate diets enriched with monoterpenes (Powell and Raffa, 1999; Broderick et al., 2004) and this is likely due to association with *Rhodococcus* gut bacteria which are able to degrade monoterpenes at high alkalinity (van der Vlugt and van der Werf, 2001).

Biosynthesis of protease inhibitors is a further means of defense employed by plants that impairs plant protein digestion by herbivorous insects (Jongsma and Bolter, 1997; Zhu-Salzman and Zeng, 2015). Bacteria-derived proteases may counter-balance the inhibition of insect-derived proteases sensitive to plant protease inhibitors. For example, the velvetbean caterpillar (*Anticarsia gemmatalis*) feeds on soy bean, which is known to possess high amounts of protease inhibitors that act as anti-herbivory defenses (Carlini and Grossi-de-Sá, 2002; Oliveira et al., 2005; da Silva Fortunato et al., 2007). This moth may use gut bacteria from the genera *Bacillus, Enterococcus*, and *Staphylococcus* to overcome these defenses as they have protease activity (Visotto et al., 2009). Characterizing gut bacterial proteases and confirming their resistance against plant-derived protease inhibitors may allow the development of analogs of protease inhibitors that target bacterial proteases, which could be used for pest control (Visotto et al., 2009; Pilon et al., 2013).

Induction of plant defenses by chewing insects may be counteracted by orally released bacteria into plant wounds, as found in the Colorado potato beetle (*Leptinotarsa decemlineata*) (Chung et al., 2013). So far, no lepidopteran species are known to orally release bacteria that inhibit induced plant defenses. On the contrary, a recent study by Wang et al. (2017) showed that gut*-*associated *Enterococcus ludwigii* of field-collected *Helicoverpa zea* caterpillars indirectly increase anti-herbivore defense of tomato plants attacked by this insect. As suggested by these authors, this difference in effects of coleopteran- and lepidopteran-associated bacteria on a plant’s anti-herbivore defense may be due to differences in the feeding modes between beetle and lepidopteran larvae. The latter ones release saliva into the wound, but hardly any bacteria-containing regurgitant, whereas the former ones release regurgitants that can contain oral and/or foregut bacteria. Another interesting example revealed that *Enterococcus* bacteria in *H. zea* larvae were found to promote increased release of salivary elicitors such as glucose oxidase leading to enhanced anti-herbivore defenses and a decrease in weight of *H. zea* larvae. Wang and co-authors suggest that field-collected *H. zea* larvae harbor this bacterium, because it might facilitate metabolism of plant toxins, but comes at the cost of triggering plant defenses.

In summary, although it is well known that insects have evolved many adaptations to counteract plant toxic defenses (Dobler et al., 2011; Heckel, 2014; Vogel et al., 2014), the composition of the bacterial community is increasingly recognized as having a great effect on how an herbivorous insect can cope with plant toxins (Mason et al., 2014). Since gut bacteria can either withstand or detoxify plant toxins (Douglas, 2009), it seems logical to speculate that the bacterial community in an insect’s gut can determine whether the insect can survive by feeding on plant tissues that are marinated with potentially toxic chemical compounds (Hammer et al., 2014).

### Intracellular Symbiotic Bacteria

Primary endosymbionts living intracellularly in specialized host cells (bacteriocytes) are well-known to establish mutualistic relationships with insects feeding on phloem, blood, or other diets with severe deficiencies in essential amino acids and/or vitamins (Douglas, 1998). However, vertically transmitted secondary endosymbionts are present across a broad range of insect taxa with diverse feeding habits, and they are known to exert different effects on their host (Weinert et al., 2015). Persistent maternal transmission of bacterial endosymbionts in lepidopterans has been demonstrated only for two symbionts: *Wolbachia* and *Spiroplasma* (Jiggins et al., 2000b; Narita et al., 2007; Ahmed et al., 2015). Several moth and butterfly species are colonized by these endosymbionts that infect the reproductive tissue and manipulate the host’s physiology to enhance their own transmission (Jiggins et al., 2000a; Hiroki et al., 2002; Kageyama et al., 2002; Dyson and Hurst, 2004; Tagami and Miura, 2004; Ahmed et al., 2015).

About 80% of lepidopteran species have been estimated to be infected by *Wolbachia* (Ahmed et al., 2015). A recent study detected a high diversity of *Wolbachia* strains in Lepidoptera comprising a total of 90 different strains (Ahmed et al., 2016). The mean infection prevalence (proportion of infection within a population) has been reported to be about 27% in Lepidoptera (Ahmed et al., 2015). A co-phylogenetic analysis of lepidopteran species and their associated *Wolbachia* strains revealed weak congruence, as closely related lepidopteran host taxa harbored distantly related *Wolbachia* strains (Ahmed et al., 2016). This finding suggests horizontal transmission, not only between individuals of the same species but also between different lepidopteran groups and probably between Lepidoptera and other insect orders.

The most common effect of *Wolbachia* infection is reproductive as it often shifts the sex ratio in favor of females. In Lepidoptera, sex ratio distortion associated to this symbiont is achieved by (1) male-killing in *Hypolimnas bolina* (killing of male embryo) (Dyson and Hurst, 2004; Charlat et al., 2007), *Acraea encedon*, and *A. encedana* (Jiggins et al., 1998; Jiggins et al., 2000a,b), (2) feminization of genetic males in *Eurema hecabe* (Hiroki et al., 2002), *Ostrinia scapulalis* (Fujii et al., 2001), and *O. furnacalis* (Kageyama et al., 2002), and (3) cytoplasmic incompatibility (gametes being unable to form viable offspring) in *Cadra cautella, Ephestia kuehniella*, and *H. bolina* (Sasaki et al., 2002; Hornett et al., 2008). In *Danaus chrysippus*, sex ratio distortion is caused by the intracellular bacterium *Spiroplasma* that induces male-killing (Jiggins et al., 2000b).

From the bacterial point of view, sex ratio distortion is an efficient strategy that promotes rapid spread in insect populations, because it results in a higher number of female offspring in *Wolbachia*-infected vs. uninfected females, thereby driving the infection into the population. The spread of male-killing *Wolbachia* in a population can be very successful, as has been shown in a population of the moth *A. encedana*, in which 95% of females were infected with *Wolbachia* (Jiggins et al., 2000a).

Although *Wolbachia* is mostly known for its role in sex ratio distortion, other effects are also known. *Wolbachia* infection can, in certain cases, lead to increased longevity and fecundity of the host. This may be caused, for example, through provisioning of riboflavin (Moriyama et al., 2015). *Wolbachia* may impact on the host’s behavior and the host’s immunity against entomopathogens (Zug and Hammerstein, 2015), and it can even manipulate the physiology of the plant in favor of its lepidopteran host (Kaiser et al., 2010). The leaf miner moth *Phyllonorycter blancardella* possesses a *Wolbachia* strain affecting cytokinin levels of apple tree leaf tissues. The cytokinin manipulation promotes plant nutrient mobility and delays senescence in those patches where the caterpillars feed on (Body et al., 2013). These effects result in the formation of photosynthetically active patches that allow larval development in otherwise decaying leaves (“green island phenotype”).

### Bacterial Symbionts and the Control of Lepidopteran Pests

Many pests have developed resistance to a great variety of pesticides, and although insect pesticide resistance is believed to be based on the genetic repertoire of the insect, recent studies have shown the potential role of bacterial symbionts in developing such resistance (van den Bosch and Welte, 2017). The bean bug *R. pedestris* harbors *Burkholderia* bacteria which can degrade an organophosphate pesticide (fenitrothion), thus conferring resistance to the host (Kikuchi et al., 2012). Studying bacterial symbionts in important lepidopteran pests such as *P. xylostella* should be of special relevance. *P. xylostella* is a worldwide pest known to have developed insecticide resistance, causing 4–5 billion dollars of damage per year (Baxter et al., 2005; Zalucki et al., 2012; Xia et al., 2013). A link between insecticide resistance and abundance of certain bacteria in the larval midgut of *P. xylostella* was found (Xia et al., 2013). The biological control agent *B. thuringiensis* (*Bt*) has been efficiently used for more than a decade against important lepidopteran pests, but resistance against the toxin produced by this microbe has also recently evolved. A potential role of gut bacteria in reducing larval mortality after exposure to *Bt* was reported for *P. xylostella* (Raymond et al., 2009). Also, mortality of the cotton bollworm, *H. armigera*, declined across generations exposed to *Bt*, whereas antibiotic-treated lines did not develop resistance (Paramasiva et al., 2014).

These studies suggest that gut bacteria are important in the evolution of *Bt* resistance, and thus elucidating the role of bacterial symbionts of lepidopterans in this context might help developing improved methods of biological control. Increasing evidence suggests that manipulation of the microbiome could reduce the abundance of pest insects in agriculture and forestry or limit disease-vectoring activity of insects (Douglas, 2007; Berasategui et al., 2016). However, all the manipulative strategies suggested so far might not only target the pest insect’s microbiome, but may also significantly affect the microbiota associated with other organisms present in the system. Genetic transformation of bacteria to impact the phenotype of the host (paratransgenesis) (Douglas, 2007) entails the risk of horizontal gene transfer to other bacteria in the ecosystem, because of the easy exchange of DNA sequences among environmental microbiota (e.g., Vos et al., 2015). Hence, development of such techniques and their use other than in laboratory research will first require very careful ecological risk assessment studies.

### Conclusions and Open Questions for Future Research

In many insect taxa, coevolution between hosts and their beneficial symbionts has been shown to broaden the ecological niches that can be colonized by the host. In Lepidoptera, such host–symbiont coevolution has not been demonstrated, because most studies have found little evidence of a core bacterial community with functional relevance in this order. However, the acquisition and transfer of some persistent bacterial members has been reported in several species, in spite of the harsh physiological conditions of the lepidopteran larval gut and the change of ecological niches between juvenile and adult stage. The high variability of the lepidopteran gut microbiome implies on the one hand that Lepidopterans do not rely on a fixed beneficial microbiota that is present in each generation. On the other hand, such variability may also imply the chance of harboring a very dynamic microbiome that allows their hosts to adapt to changing conditions including changes in abiotic conditions, food resources, and risk of natural enemy attack.

The factors leading to the evolutionary success of the highly diverse lepidopteran taxon are still unclear. According to Hammer et al. (2017), independence of microbes may have resulted in high diversification rates and lead to an extraordinary diversity and abundance of Lepidoptera. A lack of a vertically transmitted core microbiome that dictates host plant use, in combination with an ancient horizontal transfer of genes originating from bacteria (Wybouw et al., 2014) could be thus possible reasons for their success. The latter explanation has been shown to precede the diversification of phasmids (stick and leaf insects) (Shelomi et al., 2016a). While microbial symbiosis can provide novel ecological functions, Hammer and co-workers argue that dependence on symbionts might increase extinction risks because insects are constraint in their diet breadth and less able to switch to new food plants. It is therefore possible that independence of symbiosis might have facilitated switching to different host plants and promote diversification. In fact, the most species-rich superfamily of Lepidoptera, Noctuoidea, consists of many polyphagous species, among them numerous agricultural pests (Mitchell et al., 2006). Each host plant switch confronts a lepidopteran individual with a novel environment and novel microbiome present on the host plant, which may lead to the symbiont community of caterpillars being often dominated by leaf-associated bacteria that are taken up from the host plant (Hammer et al., 2017). More studies are needed to understand the relationship between plant and lepidopteran microbiomes and their role in host plant shifts, and diversification. These studies should also consider plant-induced defenses and explore how bacteria that originate from caterpillar frass or salivary regurgitants affect plant physiology as found in beetles by Chung et al. (2013).

The microbiome of moths and butterflies may not only be shaped by their interactions with plants, but also by interactions with antagonists like pathogens, predators, and parasites. Defensive symbioses are known in many animal taxa (Florez et al., 2015), and it is likely that they also exist in Lepidoptera. Defensive symbionts are often facultative, and under laboratory conditions they are likely to get lost because the pressure imposed by natural enemies is lacking. Detection of defensive symbionts in natural lepidopteran populations is therefore a challenge for future research.

Studies on individuals from the field are of great significance in order to distinguish between ecologically important bacteria colonizing lepidopterans in their natural habitats and bacteria that are the product of laboratory rearing conditions (compare: Hammer et al., 2014, 2017; Staudacher et al., 2016). There is a strong selection pressure when animals are reared under laboratory conditions. This can result in the loss of traits, including relationships with facultative symbiotic bacteria, which may have an effect that is only beneficial in natural populations.

In a nutshell, to gain a deeper understanding of the mechanisms by which Lepidoptera-associated microbes affect host traits, ecological, microbiological, and molecular approaches are needed. This knowledge will provide fundamental insights into host–microbe interactions in one of the most speciose animal groups on the planet, and may ultimately lead to a better control of important agricultural pests.

## Author Contributions

LRPV designed the first draft of this review article and performed the data analyses presented in the figures. NEF and MH contributed to the concept and structure of the review, and all authors contributed to the writing, revising, and editing of the paper.

## Conflict of Interest Statement

The authors declare that the research was conducted in the absence of any commercial or financial relationships that could be construed as a potential conflict of interest.
