# Supplementary material for: Bacterial Symbionts in Lepidoptera: Their Diversity, Transmission, and Impact on the Host
Source: Front Microbiol. 2018 Mar 27;9:556. doi: 10.3389/fmicb.2018.00556 (PMC5881003; doi:10.3389/fmicb.2018.00556)
Supplement: Supplementary file 1 [file Table_1.DOCX]

Supplementary Material

**Bacterial symbionts in Lepidoptera:**

**Their diversity, transmission and impact on the host**

**Luis R. Paniagua Voirol^1^, Enric Frago^2^, Martin Kaltenpoth^3^, Monika Hilker^1^, Nina E. Fatouros^4*^**

***Correspondence:**Nina E. Fatouros

[nina.fatouros@wur.nl](mailto:nina.fatouros@wur.nl)

**Supplementary Table 1.** List of studies used for the comparison of bacterial communities in Lepidoptera. Lepidoptera species are grouped by family. Data is focused on the presence and absence of bacteria types without taken into account their relative abundance. Lepidopteran life stage (*A*=adults, *E*=eggs, *L*=larvae, *P*=pupae), diet and screening methodology (*CB*=cultured based, *CS*=cloning of 16S rRNA gene+sequencing *HS*=high-throughput sequencing) is described per study. The list considers 30 lepidopterans from diverse ecosystems around the globe, including some model organisms, and important agricultural pests. *NA*= identification level not reported.

|  |  |  |  | **Reported bacteria** | | | |  |
| --- | --- | --- | --- | --- | --- | --- | --- | --- |
| **Lepidopteran** | **Stage** | **Diet** | **Method** | **Phylum/Class** | **Family** | **Genus** | **Species** | **Ref.** |
| **Bombycidae** | | | | | | | | |
| ***Bombyx mori*** | L | *Morus* sp. | CB | Firmicutes | Bacillaceae | *Bacillus* | *Bacillus circulans* |  |
|  |  |  |  | Gammaproteobacteria | Aeromonadaceae | *Aeromonas* | *Aeromonas* |  |
|  |  |  |  |  | Enterobacteriaceae | *Citrobacter* | *Citrobacter freundii* |  |
|  |  |  |  |  |  | *Enterobacter* | *Entrobacter* |  |
|  |  |  |  |  |  | *Erwinia* | *Erwinia* |  |
|  |  |  |  |  |  | *Escherichia* | *Escherichia coli* |  |
|  |  |  |  |  |  | *Klebsiella* | *Klebsiella pneumoniae* |  |
|  |  |  |  |  |  | *Serratia* | *Serratia liquefaciens* |  |
|  |  |  |  |  |  | *Proteus* | *Proteus vulgaris* |  |
|  |  |  |  |  | Pseudomonadaceae | *Pseudomonas* | *Pseudomonas fluorescens* |  |
|  |  |  |  |  |  |  | *Pseudomonas aeruginosa* | (Anand et al., 2010) |
| **Cossidae** | | | | | | | | |
| ***Comadia redtenbacheri*** | L | *Agave salmiana* | CB | Actinobacteria | Corynebacteriaceae | *Corynebacterium* | *Corynebacterium variabile* |  |
|  |  |  |  |  | Gordoniaceae | *Gordonia* | *NA* |  |
|  |  |  |  |  | Micrococcaceae | *Arthrobacter* | *NA* |  |
|  |  |  |  |  | Micrococcaceae | *Micrococcus* | *NA* |  |
|  |  |  |  | Firmicutes | Paenibacillaceae | *Paenibacillus* | *NA* |  |
|  |  |  |  |  | Bacillaceae | *Bacillus* | *Bacillus cereus* |  |
|  |  |  |  |  |  |  | *Bacillus safensis* |  |
|  |  |  |  |  |  |  | *Bacillus pseudomycoides* |  |
|  |  |  |  |  | Enterococcaceae | *Enterococcus* | *NA* |  |
|  |  |  |  | Gammaproteobacteria | Pseudomonadaceae | *Pseudomonas* | *NA* |  |
|  |  |  |  |  | Moraxellaceae | *Acinetobacter* | *Acinetobacter calcoaceticus* | (Hernández-Flores et al., 2015) |
| **Crambidae** | | | | | | | | |
| ***Acentria ephemerella*** | L | *Myriophyllum spicatum Potamogeton perfoliatus* | CB | Actinobacteria | Gordoniaceae | *Gordonia* | *NA* |  |
|  |  |  |  |  | Microbacteriaceae | *Leucobacter* | *NA* |  |
|  |  |  |  |  | Tsukamurellaceae | *Tsukamurella* | *NA* |  |
|  |  |  |  |  | Nocardiaceae | *Rhodococcus* | *NA* |  |
|  |  |  |  | Alphaproteobacteria | Bradyrhizobiaceae | *Afipia* | *NA* |  |
|  |  |  |  |  | Caulobacteraceae | *Brevundimonas* | *NA* |  |
|  |  |  |  |  | Sphingomonadaceae | *Sphingomonas* | *NA* |  |
|  |  |  |  | Bacteroidetes | Flavobacteriaceae | *Chryseobacterium* | *NA* |  |
|  |  |  |  | Betaproteobacteria | Comamonadaceae | *Comamonas* | *NA* |  |
|  |  |  |  | Firmicutes | Bacillaceae | *Bacillus* | *NA* |  |
|  |  |  |  | Gammaproteobacteria | Moraxellaceae | *Acinetobacter* | *NA* |  |
|  |  |  |  |  | Aeromonadaceae | *Aeromonas* | *NA* |  |
|  |  |  |  |  | Enterobacteriaceae | *Enterobacter* | *NA* |  |
|  |  |  |  |  | Pseudomonadaceae | *Pseudomonas* | *NA* | (Walenciak et al., 2002) |
| ***Diatraea saccharalis*** | L | *Saccharum officinarum* | CB | Actinobacteria | Microbacteriaceae | *Microbacterium* | *NA* |  |
|  |  |  |  | Firmicutes | Bacillaceae | *Bacillus* | *NA* |  |
|  |  |  |  |  | Enterococcaceae | *Enterococcus* | *NA* |  |
|  |  |  |  | Gammaproteobacteria | Enterobacteriaceae | *Klebsiella* | *NA* |  |
|  |  |  |  |  | Xanthomonadaceae | *Stenotrophomonas* | *NA* | (Dantur et al., 2015) |
| ***Ostrinia nubilalis*** | L | *Zea mays* | CB | Actinobacteria | Corynebacteriaceae | *Corynebacterium* | *Corynebacterium glutamicum* |  |
|  |  |  |  |  | Microbacteriaceae | *Leucobacter* | *NA* |  |
|  |  |  |  |  |  |  | *Leucobacter iarius* |  |
|  |  |  |  |  |  |  | *Leucobacter komagatae* |  |
|  |  |  |  |  |  |  | *Leucobacter luti* |  |
|  |  |  |  |  |  |  | *Leucobacter tardus* |  |
|  |  |  |  |  |  | *Microbacterium* | *Microbacterium flavescens* |  |
|  |  |  |  |  |  |  | *Microbacterium testaceum* |  |
|  |  |  |  |  |  |  | *Microbacterium thalassium* |  |
|  |  |  |  |  |  |  | *Microbacterium paraoxydans* |  |
|  |  |  |  |  |  |  | *NA* |  |
|  |  |  |  |  |  |  | *Microbacterium testaceum* |  |
|  |  |  |  | Alphaproteobacteria | Caulobacteraceae | *Brevundimonas* | *NA* |  |
|  |  |  |  |  |  |  | *Brevundimonas aurantiaca* |  |
|  |  |  |  |  | Brucellaceae | *Ochrobactrum* | *NA* |  |
|  |  |  |  |  |  |  | *Ochrobactrum pseudogrignonense* |  |
|  |  |  |  | Bacteroidetes | Flavobacteriaceae | *Chryseobacterium* | *Chryseobacterium formosense* |  |
|  |  |  |  | Betaproteobacteria | Alcaligenaceae | *Alcaligenes* | *Alcaligenes faecalis* |  |
|  |  |  |  |  | Comamonadaceae | *Variovorax* | *Variovorax paradoxus* |  |
|  |  |  |  |  |  |  | *NA* |  |
|  |  |  |  | Firmicutes | Bacillaceae | *Bacillus* | *Bacillus megaterium* |  |
|  |  |  |  |  | Paenibacillaceae | *Paenibacillus* | *Paenibacillus amylolyticus* |  |
|  |  |  |  |  |  |  | *NA* |  |
|  |  |  |  | Gammaproteobacteria | Moraxellaceae | *Acinetobacter* | *NA* |  |
|  |  |  |  |  | Pseudomonadaceae | *Pseudomonas* | *Pseudomonas aeruginosa* |  |
|  |  |  |  |  |  |  | *Pseudomonas fluorescens* |  |
|  |  |  |  |  | Enterobacteriaceae | *Serratia* | *Serratia marcescens* |  |
|  |  |  |  |  |  |  | *Serratia nematodiphila* |  |
|  |  |  |  |  |  |  | *Serratia marcescens* | (Secil et al., 2012) |
| **Erebidae** | | | | | | | | |
| ***Lymantria dispar*** | L | Artificial diet, *Quercus alba, Larix laricina, Populus tremuloides, Salix fragilis* | CB, HS | Actinobacteria | Microbacteriaceae | *Microbacterium* | *NA* |  |
|  |  |  |  |  | Micrococcaceae | *Micrococcus* | *NA* |  |
|  |  |  |  |  | Nocardiaceae | *Rhodococcus* | *NA* |  |
|  |  |  |  | Alphaproteobacteria | Rhizobiaceae | *Agrobacterium* | *NA* |  |
|  |  |  |  | Bacteroidetes | Flavobacteriaceae | *Flavobacteria* | *NA* |  |
|  |  |  |  | Firmicutes | Bacillaceae | *Bacillus* | *NA* |  |
|  |  |  |  |  | Enterococcaceae | *Enterococcus* | *Enterococcus faecalis* |  |
|  |  |  |  |  |  |  | *NA* |  |
|  |  |  |  |  | Paenibacillaceae | *Paenobacillus* | *NA* |  |
|  |  |  |  |  | Staphylococcaceae | *Staphylococcus* | *Staphylococcus lentus* |  |
|  |  |  |  |  |  |  | *Staphylococcus cohnii* |  |
|  |  |  |  |  |  |  | *Staphylococcus xylosus* |  |
|  |  |  |  | Gammaproteobacteria | Enterobacteriaceae | *Enterobacter* | *NA* |  |
|  |  |  |  |  | Enterobacteriaceae | *Pantoea* | *Pantoea agglomerans* |  |
|  |  |  |  |  | Pseudomonadaceae | *Pseudomonas* | *Psuedomonas putida* |  |
|  |  |  |  |  |  |  | *NA* |  |
|  |  |  |  |  | Enterobacteriaceae | *Serratia* | *Serratia marcescens* | (Broderick et al., 2004) |
| **Gelechiidae** |  |  |  |  |  |  |  |  |
| ***Phthorimaea operculella*** | L | Variety of crops | CB | Betaproteobacteria | Alcaligenaceae | *Alcaligenes* | *Alcaligenes faecalis* |  |
|  |  |  |  | Firmicutes | Bacillaceae | *Bacillus* | *NA* |  |
|  |  |  |  |  | Staphylococcaceae | *Staphylococcus* | *Staphylococcus sciuri* |  |
|  |  |  |  |  | Enterococcaceae | *Enterococcus* | *Enterococcus mundtii* |  |
|  |  |  |  |  |  |  | *Enterococcus casseiflavus* |  |
|  |  |  |  | Gammaproteobacteria | Enterobacteriaceae | *Enterobacter* | *NA* |  |
|  |  |  |  |  |  | *Pantoea* | *Pantoea agglomerans* |  |
|  |  |  |  |  | Pseudomonadaceae | *Pseudomonas* | *Pseudomonas fluorescens* |  |
| ***Sitotroga cerealella*** | L | Variety of crops | CB | Firmicutes | Staphylococcaceae | *Staphylococcus* | *Staphylococcus succinus* |  |
|  |  |  |  |  | Enterococcaceae | *Enterococcus* | *NA* | (Sevim et al., 2016) |
| **Hepialidae** | | | | | | | | |
| ***Hepalius gonggaensis*** | L | Not reported (caught from wild natural environment) | HS | Actinobacteria | Solirubrobacteraceae | *Solirubrobacter* | *NA* |  |
|  |  |  |  | Firmicutes | Carnobacteriaceae | *Carnobacterium* | *NA* |  |
|  |  |  |  |  | Staphylococcaceae | *Staphylococcus* | *Staphylococcus kloosii* |  |
|  |  |  |  | Gammaproteobacteria | Enterobacteriaceae | *Enterobacteriaceae* | *NA* |  |
|  |  |  |  |  | Pseudomonadaceae | *Pseudomonas* | *NA* |  |
|  |  |  |  |  |  |  | *Pseudomonas fluorescens* |  |
|  |  |  |  |  | Enterobacteriaceae | *Rahnella* | *NA* | (Yu et al., 2008) |
| **Noctuidae** | | | | | | | | |
| ***Anticarsia gemmatalis*** | L | Soybean Artificial diet | CB | Firmicutes | Bacillaceae | *Bacillus* | *Bacillus cereus* |  |
|  |  |  |  |  |  |  | *Bacillus subtilis* |  |
|  |  |  |  |  | Enterococcaceae | *Enterococcus* | *Enterococcus gallinarum* |  |
|  |  |  |  |  |  |  | *Enterococcus mundtii* |  |
|  |  |  |  |  | Staphylococcaceae | *Staphylococcus* | *Staphylococcus xylosis* | (Visôtto et al., 2009) |
| ***Brithys crini*** | L | *Pancratium maritimum* | HS | Actinobacteria | Propionibacterineae | *Nocardioides* | *NA* |  |
|  |  |  |  |  | Streptomycetaceae | *Streptomyces* | *NA* |  |
|  |  |  |  | Alphaproteobacteria | Rhizobiaceae | *Agrobacterium* | *NA* |  |
|  |  |  |  |  | Sphingomonadaceae | *Sphingomonas* | *NA* |  |
|  |  |  |  | Betaproteobacteria | Burkholderiaceae | *NA* | *NA* |  |
|  |  |  |  | Firmicutes | Enterococcaceae | *NA* | *NA* |  |
|  |  |  |  |  | Bacillaceae | *NA* | *NA* |  |
|  |  |  |  |  | Staphylococcaceae | *Staphylococcus* | *NA* |  |
|  |  |  |  | Gammaproteobacteria | Enterobacteriaceae | *Citrobacter* | *NA* |  |
|  |  |  |  |  |  | *Erwinia* | *NA* |  |
|  |  |  |  |  |  | *NA* | *NA* |  |
|  |  |  |  |  | Xanthomonadaceae | *NA* | *NA* |  |
|  |  |  |  |  | Pseudomonadaceae | *Pseudomonas* | *NA* | (Vilanova et al., 2016) |
| ***Busseola fusca*** | L | *Zea mays* | CB | Actinobacteria | Micrococcaceae | *Arthrobacter* | *Arthrobacter oxydans* |  |
|  |  |  |  |  | Microbacteriaceae | *Leucobacter* | *Leucobacter chromiiresistens* |  |
|  |  |  |  |  |  |  | *Leucobacter tardus* |  |
|  |  |  |  |  |  | *Microbacterium* | *Microbacterium testaceum* |  |
|  |  |  |  | Alphaproteobacteria | Caulobacteraceae | *Brevundimonas* | *Brevundimonas naejangsanensis* |  |
|  |  |  |  |  |  | *Caulobacter* | *Caulobacter segnis* |  |
|  |  |  |  | Betaproteobacteria | Alcaligenaceae | *Achromobacter* | *Achromobacter spanius* |  |
|  |  |  |  | Firmicutes | Bacillaceae | *Bacillus* | *Bacillus licheniformis* |  |
|  |  |  |  |  |  |  | *Bacillus simplex* |  |
|  |  |  |  |  |  |  | *Bacillus thuringienisis* |  |
|  |  |  |  |  | Paenibacillaceae | *Brevibacillus* | *Brevibacillus borstelensis* |  |
|  |  |  |  |  | Enterococcaceae | *Enterococcus* | *Enterococcus casselifalvus* |  |
|  |  |  |  |  | Streptococcaceae | *Lactococcus* | *Lactococcus lactis* |  |
|  |  |  |  |  | Planococcaceae | *Planomicrobium* | *Planomicrobium chinense* |  |
|  |  |  |  |  |  | *Solibacillus* | *Solibacillus silvestris* |  |
|  |  |  |  |  | Staphylococcaceae | *Staphylococcus* | *Staphylococcus haemolyticus* |  |
|  |  |  |  | Gammaproteobacteria | Enterobacteriaceae | *Klebsiella* | *Klebsiella variicola* |  |
|  |  |  |  |  | Pseudomonadaceae | *Pseudomonas* | *Pseudomonas geniculata* |  |
|  |  |  |  |  |  |  | *Pseudomonas mosselii* |  |
|  |  |  |  |  |  |  | *Pseudomonas protegens* | (Snyman et al., 2016) |
| ***Calyptra thalictri*** | A | Not reported (caught from wild natural environment) | CS, HS | Alphaproteobacteria | Rhizobiaceae | *Rhizobium* | *NA* |  |
|  |  |  |  |  |  | *Sinorhizobium* | *NA* |  |
|  |  |  |  | Betaproteobacteria | Alcaligenaceae | *Alcaligenes* | *NA* |  |
|  |  |  |  | Gammaproteobacteria | Enterobacteriaceae | *Klebsiella* | *NA* | (Zaspel and Hoy, 2008) |
| ***Helicoverpa armigera*** | L | *Zea mays* | HS | Actinobacteria | Actinomycetaceae | *Actinomyces* | *NA* |  |
|  |  |  |  |  | Corynebacteriaceae | *NA* | *NA* |  |
|  |  |  |  |  | Micrococcaceae | *NA* | *NA* |  |
|  |  |  |  |  | Propionibacteriaceae | *NA* | *NA* |  |
|  |  |  |  |  | Micrococcaceae | *Micrococcus* | *NA* |  |
|  |  |  |  |  | Nocardiaceae | *Rhodococcus* | *Rhodococcus baikonurensis* |  |
|  |  |  |  |  | Propionibacteriaceae | *Propionibacterium* | *NA* |  |
|  |  |  |  | Alphaproteobacteria | Acetobacteraceae | *Gluconobacter* | *Gluconobacter cerinus* |  |
|  |  |  |  |  | Caulobacteraceae | *Brevundimonas* | *Brevundimonas diminuta* |  |
|  |  |  |  |  |  | *Caulobacter* | *NA* |  |
|  |  |  |  |  | Sphingomonadaceae | *Sphingomonas* | *NA* |  |
|  |  |  |  |  | Caulobacteraceae | *NA* | *NA* |  |
|  |  |  |  |  | Rhodobacteraceae | *NA* | *NA* |  |
|  |  |  |  |  |  | *Paracoccus* | *Paracoccus carotinifaciens* |  |
|  |  |  |  | Bacteroidetes | Flavobacteriaceae | *Chryseobacterium* | *Chryseobacterium meningosepticum* |  |
|  |  |  |  |  |  | *Flavobacterium* | *NA* |  |
|  |  |  |  |  |  | *NA* | *NA* |  |
|  |  |  |  |  | Flexibacteriaceae | *NA* | *NA* |  |
|  |  |  |  |  | Sphingobacteriaceae | *NA* | *NA* |  |
|  |  |  |  | Betaproteobacteria | Alcaligenaceae | *Bordetella* | *NA* |  |
|  |  |  |  |  | Burkholderiaceae | *Burkholderia* | ***NA*** |  |
|  |  |  |  |  | Comamonadaceae | *Comamonas* | *NA* |  |
|  |  |  |  |  |  |  | *Comamonas testosteroni* |  |
|  |  |  |  | Deinococcus-Thermus | Deinococcaceae | *Deinococcus* | *NA* |  |
|  |  |  |  | Epsilonproteobacteria | Campylobacteraceae | *NA* | *NA* |  |
|  |  |  |  | Firmicutes | Aerococcaceae | *NA* | *NA* |  |
|  |  |  |  |  | Bacillaceae | *NA* | *NA* |  |
|  |  |  |  |  | Carnobacteriaceae | *Carnobacterium* | *Carnobacterium maltaromaticum* |  |
|  |  |  |  |  | Clostridiaceae | *NA* | *NA* |  |
|  |  |  |  |  | Enterococcaceae | *Enterococcus* | *Enterococcus mundtii* |  |
|  |  |  |  |  |  |  | *Enterococcus faecium* |  |
|  |  |  |  |  |  |  | *Enterococcus casseliflavus* |  |
|  |  |  |  |  |  |  | *Enterococcus avium* |  |
|  |  |  |  |  |  |  | *Enterococcus gallinarum* |  |
|  |  |  |  |  |  | *NA* | *NA* |  |
|  |  |  |  |  | Erysipelotrichaceae | *NA* | *NA* |  |
|  |  |  |  |  | Halobacillaceae | *NA* | *NA* |  |
|  |  |  |  |  | Lachnospiraceae | *NA* | *NA* |  |
|  |  |  |  |  | Lactobacillaceae | *NA* | *NA* |  |
|  |  |  |  |  | Leuconostocaceae | *Leuconostoc* | *Leuconostoc citreum* |  |
|  |  |  |  |  | Peptostreptococcaceae | *Clostridium* | *NA* |  |
|  |  |  |  |  | Staphylococcaceae | *Staphylococcus* | ***NA*** |  |
|  |  |  |  |  | Streptococcaceae | *Lactococcus* | *NA* |  |
|  |  |  |  |  |  |  | *Lactococcus lactis* |  |
|  |  |  |  |  |  | *NA* | *NA* |  |
|  |  |  |  | Gammaproteobacteria | Alteromonadaceae | *NA* | *NA* |  |
|  |  |  |  |  | Catabacter | *NA* | *NA* |  |
|  |  |  |  |  | Enterobacteriaceae | *Aquamonas* | *Aquamonas fontana* |  |
|  |  |  |  |  |  | *Enterobacter* | *NA* |  |
|  |  |  |  |  |  | *Escherichia* | *Escherichia coli* |  |
|  |  |  |  |  |  | *NA* | *NA* |  |
|  |  |  |  |  | Moraxellaceae | *Acinetobacter* | *Acinetobacter anitratus* |  |
|  |  |  |  |  |  |  | *NA* |  |
|  |  |  |  |  |  |  | *Acinetobacter venetianus* |  |
|  |  |  |  |  | Pseudomonadaceae | *Pseudomonas* | *Pseudomonas mevalonii* |  |
|  |  |  |  |  | Xanthomonadaceae | *Stenotrophomonas* | *Stenotrophomonas maltophilia* |  |
|  |  |  |  | NA | Annamoxales | *NA* | *NA* |  |
|  |  |  |  |  | NA | *NA* | *Phenanthrene-degrading bacterium* |  |
|  |  |  |  | Planctomycetes | Planctomycetaceae | *NA* | *NA* |  |
|  |  |  |  | Thermodesulfobacteria | Thermodesulfobacteriaceae | *NA* | *NA* | (Tang et al. 2012;Xiang et al. 2006) |
| ***Heliothis virescens*** | A, E, L | Artificial diet, *Gossypium hirsutum, Cicer arietinum, Nicotiana attenuata (larvae),* Sugar solution (adults) | HS | Acidobacteria | NA | *NA* | *NA* |  |
|  |  |  |  | Actinobacteria | Mycobacteriaceae | *Mycobacterium* | *NA* |  |
|  |  |  |  | Alphaproteobacteria | Acetobacteraceae | *Asaia* | *Asaia* |  |
|  |  |  |  |  | Methylobacteriaceae | *Methylobacterium* | *NA* |  |
|  |  |  |  | Betaproteobacteria | Comamonadaceae | *Roseateles* | *NA* |  |
|  |  |  |  | Firmicutes | Enterococcaceae | *Enterococcus* | *NA* |  |
|  |  |  |  |  | Paenibacillaceae | *Paenibacillus* | *NA* |  |
|  |  |  |  | Gammaproteobacteria | Moraxellaceae | *Acinetobacter* | *NA* |  |
|  |  |  |  |  | Enterobacteriaceae | *Enterobacter* | *NA* |  |
|  |  |  |  |  |  | *Serratia* | *NA* |  |
|  |  |  |  |  |  | *NA* | *NA* |  |
|  |  |  |  |  |  | *Enterobacter* | *NA* |  |
|  |  |  |  |  | Pseudomonadaceae | *Pseudomonas* | *NA* |  |
|  |  |  |  |  | Xanthomonadaceae | *Stenotrophomonas* | *NA* | (Staudacher et al., 2016) |
| ***Mythimna separata*** | L | *Zea mays* | CB, CS, HS | Actinobacteria | Micrococcaceae | *Arhrobacter* | *NA* |  |
|  |  |  |  |  | Microbacteriaceae | *Frigoribacterium* | *NA* |  |
|  |  |  |  | Alphaproteobacteria | Brucellaceae | *Ochrobactrum* | *Ochrobactrum anthropi* |  |
|  |  |  |  | Betaproteobacteria | Ralstoniaceae | *Ralstonia pickettii* | *Ralstonia pickettii* |  |
|  |  |  |  | Firmicutes | Bacillaceae | *Bacillus* | *Bacillus pumilus* |  |
|  |  |  |  |  |  |  | *Bacillus safensis* |  |
|  |  |  |  |  | Enterococcaceae | *Enterococcus* | *Enterococcus faecium* |  |
|  |  |  |  |  |  |  | *Enterococcus mundtii* |  |
|  |  |  |  |  | Staphylococcaceae | *Staphylococcus* | *Staphylococcus equorum* |  |
|  |  |  |  |  |  |  | *Staphylococcus gallinarum* |  |
|  |  |  |  |  |  |  | *Staphylococcus saprophyticus* |  |
|  |  |  |  |  |  |  | *Staphylococcus sciuri* |  |
|  |  |  |  |  |  |  | *Staphylococcus xylosus* |  |
|  |  |  |  | Gammaproteobacteria | Enterobacteriaceae | *Escherichia* | *Escherichia coli* |  |
|  |  |  |  |  |  |  | *NA* |  |
|  |  |  |  |  |  | *Pantoea* | *Pantoea ananatis* |  |
|  |  |  |  |  | Pseudomonadaceae | *Pseudomonas* | *Pseudomonas aeruginosa* |  |
|  |  |  |  |  |  |  | *Pseudomonas veronii* | (He et al., 2013) |
| ***Sesamia nonagrioides*** | L | *Zea mays* | CB | Actinobacteria | Micrococcaceae | *Arthrobacter* | *Arthrobacter protophormiae* |  |
|  |  |  |  |  | Microbacteriaceae | *Microbacterium* | *Microbacterium arborescens* |  |
|  |  |  |  | Bacteroidetes | Flavobacteriaceae | *Chryseobacterium* | *Chryseobacterium indologenes* |  |
|  |  |  |  | Betaproteobacteria | Alcaligenaceae | *Achromobacter* | *Achromobacter insolitus* |  |
|  |  |  |  | Firmicutes | Bacillaceae | *Bacillus* | *Bacillus thuringiensis* |  |
|  |  |  |  |  |  |  | *Bacillus safensis* |  |
|  |  |  |  |  | Staphylococcaceae | *Staphylococcus* | *Staphylococcus sciuri* |  |
|  |  |  |  | Gammaproteobacteria | Enterobacteriaceae | *Morganella* | *Morganella morganii* |  |
|  |  |  |  |  |  | *Klebsiella* | *Klebsiella pneumoniae* |  |
|  |  |  |  |  |  | *Citrobacter* | *Citrobacter freundii* |  |
|  |  |  |  |  |  | *Enterobacter* | *Enterobacter kobei* |  |
|  |  |  |  |  |  | *Serratia* | *Serratia marcescens* | (Eski et al., 2015) |
| ***Spodoptera littoralis*** | A, E, L, P, A | Artificial diet, vegetable garden (larvae), sucrose solution (adults) | CS, HS | Acidobacteria | Acidobacteriaceae | *NA* | *NA* |  |
|  |  |  |  | Actinobacteria | Corynebacteriaceae | *NA* | *NA* |  |
|  |  |  |  |  | Micrococcaceae | *NA* | *NA* |  |
|  |  |  |  |  | Propionibacteriaceae | *NA* | *NA* |  |
|  |  |  |  |  | Thermomonosporaceae | *Thermomonospora* | *NA* |  |
|  |  |  |  |  | Microbacteriaceae | *Microbacterium* | *Microbacterium hominis* |  |
|  |  |  |  |  | Propionibacteriaceae | *Propionibacterium* | *Propionibacterium acnes* |  |
|  |  |  |  | Alphaproteobacteria | Caulobacteraceae | *NA* | *NA* |  |
|  |  |  |  |  | Rhodobacteraceae | *NA* | *Rhodobacteraceae* |  |
|  |  |  |  |  | Rhodobacteraceae | *Paracoccus* | *Paracoccus solventivorans* |  |
|  |  |  |  | Betaproteobacteria | Oxalobacteraceae | *Massilia* | *Massilia timonae* |  |
|  |  |  |  |  | Ralstoniaceae | *Ralstonia* | *NA* |  |
|  |  |  |  | Chloroflexi | Anaerolineaceae | *NA* | *NA* |  |
|  |  |  |  |  | Dehalococcoidaceae | *Dehalococcoides* | ***NA*** |  |
|  |  |  |  | Deltaproteobacteria | Desulfovibrionaceae | *NA* | *NA* |  |
|  |  |  |  | Firmicutes | Bacillaceae | *NA* | *NA* |  |
|  |  |  |  |  | Clostridiaceae | *NA* | *NA* |  |
|  |  |  |  |  | Enterococcaceae | *Enterococcus* | *Enterococcus casseliflavus* |  |
|  |  |  |  |  |  |  | *Enterococcus faecalis* |  |
|  |  |  |  |  |  |  | *Enterococcus mundtii* |  |
|  |  |  |  |  |  |  | *Enterococcus termitis* |  |
|  |  |  |  |  |  |  | *NA* |  |
|  |  |  |  |  |  | *NA* | *NA* |  |
|  |  |  |  |  | Erysipelotrichaceae | *NA* | *NA* |  |
|  |  |  |  |  | Halobacillaceae | *NA* | *NA* |  |
|  |  |  |  |  | Lachnospiraceae | *NA* | *NA* |  |
|  |  |  |  |  | Lactobacillaceae | *Lactobacillus* | *NA* |  |
|  |  |  |  |  |  | *NA* | *NA* |  |
|  |  |  |  |  |  | *Pediococcus* | *NA* |  |
|  |  |  |  |  | Leuconostocaceae | *Weissella* | *NA* |  |
|  |  |  |  |  | Peptostreptococcaceae | *Clostridium* | *Clostridium cocleatum* |  |
|  |  |  |  |  |  |  | *Clostridium piliforme* |  |
|  |  |  |  |  |  |  | *Clostridium ramosum* |  |
|  |  |  |  |  |  |  | *NA* |  |
|  |  |  |  |  | Streptococcaceae | *NA* | *Streptococcaceae* |  |
|  |  |  |  | Gammaproteobacteria | Alteromonadaceae | *NA* | *NA* |  |
|  |  |  |  |  | Enterobacteriaceae | *Citrobacter* | *NA* |  |
|  |  |  |  |  |  | *Enterobacter* | *Enterobacter asburiae* |  |
|  |  |  |  |  |  | *Escherichia* | *Escherichia coli* |  |
|  |  |  |  |  |  | *Klebsiella* | *Klebsiella pneumonia* |  |
|  |  |  |  |  |  |  | *NA* |  |
|  |  |  |  |  |  | *NA* | *NA* |  |
|  |  |  |  |  |  | *Pantoea* | *NA* |  |
|  |  |  |  |  |  |  | *Pantoea agglomerans* |  |
|  |  |  |  |  |  | *Serratia* | *NA* |  |
|  |  |  |  |  | Moraxellaceae | *Acinetobacter* | *NA* |  |
|  |  |  |  |  | Pseudomonadaceae | *Pseudomonas* | *NA* |  |
|  |  |  |  |  |  |  | *Pseudomonas oryzihabitans* |  |
|  |  |  |  |  |  |  | *Pseudomonas psychrotolerans* |  |
|  |  |  |  |  |  |  | *Pseudomonas putida* |  |
|  |  |  |  |  | Xanthomonadaceae | *Xanthomonas* | *Xanthomonas campestris* |  |
|  |  |  |  | NA | Catabacter | *NA* | *NA* |  |
|  |  |  |  |  | Flexibacteriaceae | *NA* | *NA* |  |
|  |  |  |  | Planctomycetes | Planctomycetaceae | *NA* | *NA* | (Tang et al. 2012; Chen et al. 2016 |
| ***Spodoptera litura*** | L | *Ricinus communis* | CB | Actinobacteria | Microbacteriaceae | *Microbacterium* | *Microbacterium arborescens* |  |
|  |  |  |  | Firmicutes | Enterococcaceae | *Enterococcus* | *Enterococcus casseliflavus* |  |
|  |  |  |  | Gammaproteobacteria | Enterobacteriaceae | *Enterobacter* | *Enterobacter cloacae* | (Thakur et al., 2015) |
| **Nymphalidae** | | | | | | | | |
| ***Heliconius herato*** | A, L, P, F | *Psychotria elata and Lantana* spp., (adults) *Passiflora biflora (larvae)* | HS | Alphaproteobacteria | Acetobacteraceae | *Asaia* | *NA* |  |
|  |  |  |  |  |  | *Commensalibacter* | *NA* |  |
|  |  |  |  | Firmicutes | Enterococcaceae | *Enterococcus* | *NA* |  |
|  |  |  |  |  | Streptococcaceae | *Lactococcus* | *NA* |  |
|  |  |  |  | Gammaproteobacteria | Moraxellaceae | *Acinetobacter* | *NA* |  |
|  |  |  |  |  | Enterobacteriaceae | *Enterobacter* | ***NA*** |  |
|  |  |  |  |  | Pseudomonadaceae | *NA* | *NA* | (Hammer et al., 2014) |
| **Saturniidae** | | | | | | | | |
| ***Antheraea assamensis*** | L | *Persea bombycina* | CB | Betaproteobacteria | Alcaligenaceae | *Achromobacter* | *Achromobacter marplatensis* |  |
|  |  |  |  |  | Alcaligenaceae |  | *Achromobacter xylosoxidans* |  |
|  |  |  |  | Firmicutes | Bacillaceae | *Bacillus* | *Bacillus pumilus* |  |
|  |  |  |  |  |  |  | *Bacillus subtilis* |  |
|  |  |  |  |  |  |  | *NA* |  |
|  |  |  |  |  |  | *Orinithinibacillus* | *Orinithinibacillus bavariensis* |  |
|  |  |  |  |  |  | *Paucisalibacillus* | *Paucisalibacillus globulus* |  |
|  |  |  |  |  | Staphylococcaceae | *Staphylococcus* | *Staphylococcus aureus* |  |
|  |  |  |  |  |  |  | *Staphylococcus pasteuri* |  |
|  |  |  |  |  |  |  | *Staphylococcus warneri* |  |
|  |  |  |  | Gammaproteobacteria | Pseudomonadaceae | *Pseudomonas* | *Pseudomonas aeruginosa* | (Haloi et al., 2016) |
| ***Automeris zugana*** | L, P | *Cydista heterophylla, Trigonia rugosa, Calycophyllum candidissimum, Annona purpurea, Inga vera, Quercus oleoides, Paullinia cururu, Cordia alliodora* | CB | Actinobacteria | Corynebacteriaceae | *Corynebacterium* | *Corynebacterium durum* |  |
|  |  |  |  |  |  |  | *Corynebacterium pseudotuberculosis* |  |
|  |  |  |  |  |  |  | *NA* |  |
|  |  |  |  |  | Microbacteriaceae | *Curtobacterium* | *NA* |  |
|  |  |  |  |  | Micrococcaceae | *Micrococcus* | *NA* |  |
|  |  |  |  | Firmicutes | Bacillaceae | *Bacillus* | *Bacillus mascerans* |  |
|  |  |  |  |  |  |  | *NA* |  |
|  |  |  |  |  | Leuconostocaceae | *Leuconostoc* | *Leuconostoc citreum* |  |
|  |  |  |  |  | Listeriaceae | *Listeria* | *Listeria monocytogenes* |  |
|  |  |  |  |  | Staphylococcaceae | *Staphylococcus* | *NA* |  |
|  |  |  |  |  | Staphylococcaceae |  | *Staphylococcus sciuri* |  |
|  |  |  |  | Gammaproteobacteria | Enterobacteriaceae | *Citrobacter* | *Citrobacter amalonicus* |  |
|  |  |  |  |  |  |  | *Citrobacter freundii* |  |
|  |  |  |  |  |  |  | *Citrobacter koserri* |  |
|  |  |  |  |  |  | *Enterobacter* | *Enterobacter aerogenes* |  |
|  |  |  |  |  |  |  | *Enterobacter agglomerans* |  |
|  |  |  |  |  |  |  | *Enterobacter cloacae* |  |
|  |  |  |  |  |  |  | *Enterobacter gergoviae* |  |
|  |  |  |  |  |  |  | *NA* |  |
|  |  |  |  |  |  | *Klebsiella* | *Klebsiella pneumoniae* |  |
|  |  |  |  |  | Pseudomonadaceae | *Pseudomonas* | *NA* |  |
|  |  |  |  |  |  |  | *Pseudomonas diminuta* |  |
|  |  |  |  |  |  |  | *Pseudomonas mallei* |  |
|  |  |  |  |  |  |  | *Pseudomonas syringue* |  |
|  |  |  |  |  | Enterobacteriaceae | *Serratia* | *NA* |  |
|  |  |  |  |  |  |  | *Serratia licuefaciens* |  |
|  |  |  |  |  |  |  | *Serratia marcesens* |  |
|  |  |  |  | Actinobacteria | Corynebacteriaceae | *Corynebacterium* | *Corynebacterium pyogenes* |  |
|  |  |  |  | Firmicutes | Bacillaceae | *Bacillus* | *Bacillus thuringiensis* |  |
|  |  |  |  |  | Staphylococcaceae | *Staphylococcus* | *Staphylococcus aureus* | (Sittenfeld et al., 2002;Pinto-Tomás et al., 2007) |
| ***Citheronia lobesis*** | L, E | *Spondias mombin,* Artificial diet. | CS, HS | Actinobacteria | Corynebacteriaceae | *Corynebacterium* | *NA* |  |
|  |  |  |  |  | Microbacteriaceae | *Curtobacterium* | *NA* |  |
|  |  |  |  |  | Propionibacteriaceae | *Propionibacterium* | *NA* |  |
|  |  |  |  | Alphaproteobacteria | Bradyrhizobiaceae | *Bradyrhizobium* | *NA* |  |
|  |  |  |  |  | Caulobacteraceae | *Caulobacter* | *NA* |  |
|  |  |  |  |  | Methylobacteriaceae | *Methylobacterium* | *NA* |  |
|  |  |  |  |  | Sphingomonadaceae | *Sphingomonas* | *NA* |  |
|  |  |  |  |  |  |  | *Sphingomonas mali* |  |
|  |  |  |  |  | Caulobactereaceae | *NA* | *NA* |  |
|  |  |  |  |  | Brucellaceae | *Ochrobactrum* | *NA* |  |
|  |  |  |  | Bacteroidetes | Flavobacteriaceae | *Flavobacterium* | *NA* |  |
|  |  |  |  | Betaproteobacteria | Comamonadaceae | *Acidovorax* | *NA* |  |
|  |  |  |  |  | Oxalobacteraceae | *Janthinobacterium* | *NA* |  |
|  |  |  |  |  | Ralstoniaceae | *Ralstonia* | *NA* |  |
|  |  |  |  | Firmicutes | Bacillaceae | *Bacillus* | *Bacillus subtilis* |  |
|  |  |  |  |  |  | *Bacillus* | *Bacillus cereus/thuringiensis* |  |
|  |  |  |  |  | Enterococcaceae | *Enterococcus* | *NA* |  |
|  |  |  |  |  | Staphylococcaceae | *Staphylococcus* | *NA* |  |
|  |  |  |  |  | Streptococcaceae | *Streptococcus* | *NA* |  |
|  |  |  |  | Gammaproteobacteria | Moraxellaceae | *Acinetobacter* | *NA* |  |
|  |  |  |  |  |  |  | *NA* |  |
|  |  |  |  |  | Enterobacteriaceae | *Citrobacter* | *Citrobacter freundii* |  |
|  |  |  |  |  |  | *Enterobacter* | *NA* |  |
|  |  |  |  |  |  | *Escherichia* | *Escherichia coli* |  |
|  |  |  |  |  |  | *Pantoea* | *Pantoea agglomerans* |  |
|  |  |  |  |  |  | *Serratia* | *Serratia marcescens* |  |
|  |  |  |  |  | Xanthomonadaceae | *Stenotrophomonas* | *NA* | (Pinto-Tomás et al., 2011) |
| ***Hylesia metabus*** | L | *Rhizophora mangle, Hura crepitans* | CB | Actinobacteria | Corynebacteriaceae | *Corynebacterium* | *NA* |  |
|  |  |  |  |  |  |  | *Corynebacterium urealyticum* |  |
|  |  |  |  |  | Nocardiaceae | *Rhodococcus* | *NA* |  |
|  |  |  |  | Bacteroidetes | Flavobacteriaceae | *Chryseobacterium* | *NA* |  |
|  |  |  |  | Betaproteobacteria | Alcaligenaceae | *Alcaligenes* | *Alcaligenes faecalis* |  |
|  |  |  |  | Firmicutes | Bacillaceae | *Bacillus* | *Bacillus subtilus* |  |
|  |  |  |  |  |  |  | *Bacillus megaterium* |  |
|  |  |  |  |  | Enterococcaceae | *Enterococcus* | *NA* |  |
|  |  |  |  |  | Planococcaceae | *Planococcus* | *NA* |  |
|  |  |  |  |  | Staphylococcaceae | *Staphylococcus* | *Staphylococcus gallinarum* |  |
|  |  |  |  |  |  |  | *Staphylococcus sciuri* |  |
|  |  |  |  |  |  |  | *Staphylococcus warneri* |  |
|  |  |  |  | Gammaproteobacteria | Moraxellaceae | *Acinetobacter* | *NA* |  |
|  |  |  |  |  | Enterobacteriaceae | *Enterobacter* | *NA* |  |
|  |  |  |  |  |  |  | *Enterobacter cloacae* |  |
|  |  |  |  |  |  | *Klebsiella* | *Klebsiella oxytoca* | (Osborn et al., 2002) |
| ***Rothschildia lebeau*** | L, P | Wild diet, Artificial diet and *Spondias mombin* | CB, CS, HS | Acidobacteria | Acidobacteriaceae | *NA* | *NA* |  |
|  |  |  |  | Actinobacteria | Actinoplanaceae | *Actinoplanes* | *NA* |  |
|  |  |  |  |  | Microbacteriaceae | *Aureobacterium* | *Aureobacterium testaceum* |  |
|  |  |  |  |  |  | *Curtobacterium* | *Curtobacterium albidum* |  |
|  |  |  |  |  |  |  | *Curtobacterium citreum* |  |
|  |  |  |  |  |  |  | *Curtobacterium pusillum* |  |
|  |  |  |  |  |  |  |  |  |
|  |  |  |  |  |  | *Microbacterium* | *Microbacterium laevaniformans* |  |
|  |  |  |  |  | Micrococcaceae | *Micrococcus* | *Micrococcus luteus* |  |
|  |  |  |  |  | Propionibacteriaceae | *Propionibacterium* | *NA* |  |
|  |  |  |  | Alphaproteobacteria | Rhizobiaceae | *Agrobacterium* | *Agrobacterium larrymoorei* |  |
|  |  |  |  |  |  |  | *Agrobacterium rhizogenes* |  |
|  |  |  |  |  | Caulobacteraceae | *Brevundimonas* | *Brevundimonas diminuta* |  |
|  |  |  |  |  |  |  | *Brevundimonas nasdae* |  |
|  |  |  |  |  | Bradyrhizobiaceae | *Bradyrhizobium* | *NA* |  |
|  |  |  |  |  | Caulobacteraceae | *Caulobacter* | *NA* |  |
|  |  |  |  |  | Methylobacteriaceae | *Methylobacterium* | *NA* |  |
|  |  |  |  |  | Rhizobiaceae | *Rhizobium* | *NA* |  |
|  |  |  |  |  | Sphingomonadaceae | *Sphingomonas* | *NA* |  |
|  |  |  |  |  |  |  | *Sphingomonas pruni* |  |
|  |  |  |  | Bacteroidetes | Flavobacteriaceae | *Flavobacterium* | *NA* |  |
|  |  |  |  | Betaproteobacteria | Comamonadaceae | *Comamonas* | *Comamonas terrigena* |  |
|  |  |  |  |  |  | *Curvibacter* | *Curvibacter gracilis* |  |
|  |  |  |  |  |  | *Acidovorax* | *NA* |  |
|  |  |  |  |  |  | *Comamonas* | *NA* |  |
|  |  |  |  |  | Ralstoniaceae | *Ralstonia* | *NA* |  |
|  |  |  |  | Firmicutes | Bacillaceae | *Bacillus* | *Bacillus amyloliquefaciens* |  |
|  |  |  |  |  |  |  | *Bacillus cereus/thuringiensis* |  |
|  |  |  |  |  |  |  | *Bacillus megaterium* |  |
|  |  |  |  |  |  |  | *Bacillus subtilis* |  |
|  |  |  |  |  | Carnobacteriaceae | *Carnobacterium* | *Carnobacterium divergens* |  |
|  |  |  |  |  | Staphylococcaceae | *Staphylococcus* | *NA* |  |
|  |  |  |  |  |  |  | *Staphylococcus arlettae* |  |
|  |  |  |  |  |  |  | *Staphylococcus aureus* |  |
|  |  |  |  |  |  |  | *Staphylococcus equorum* |  |
|  |  |  |  |  |  |  | *Staphylococcus haemolyticus* |  |
|  |  |  |  |  |  |  | *Staphylococcus lentus* |  |
|  |  |  |  |  |  |  | *Staphylococcus saprophyticus* |  |
|  |  |  |  |  |  |  | *Staphylococcus suarezii* |  |
|  |  |  |  |  | Streptococcaceae | *Streptococcus* | *NA* |  |
|  |  |  |  | Gammaproteobacteria | Enterobacteriaceae | *Enterobacter* | *Enterobacter cloacae* |  |
|  |  |  |  |  |  |  | *NA* |  |
|  |  |  |  |  |  | *Morganella* | *Morganella morganii* |  |
|  |  |  |  |  |  | *Escherichia* | *Escherichia coli* |  |
|  |  |  |  |  |  | *Klebsiella* | *Klebsiella planticola* |  |
|  |  |  |  |  |  |  | *Klebsiella terrigena* |  |
|  |  |  |  |  |  |  | *NA* |  |
|  |  |  |  |  |  | *Pantoea* | *Pantoea dispersa* |  |
|  |  |  |  |  |  |  | *Pantoea agglomerans* |  |
|  |  |  |  |  |  | *Proteus* | *Proteus mirabilis* |  |
|  |  |  |  |  |  | *Rahnella* | *Rhanella aquatilis* |  |
|  |  |  |  |  |  | *Serratia* | *Serratia marcescens* |  |
|  |  |  |  |  | Moraxellaceae | *Acinetobacter* | *NA* |  |
|  |  |  |  |  | Pseudomonadaceae | *Pseudomonas* | *NA* |  |
|  |  |  |  |  |  |  | *Pseudomonas maculicula* |  |
|  |  |  |  |  |  |  | *Psuedomonas stutzeri* | (Pinto-Tomás et al., 2007;Pinto-Tomás et al., 2011) |
|  |  |  |  |  | Pasteurellaceae | *Terrahaemophilus* | *Terrahaemophilus aromaticivorans* |  |
|  |  |  |  |  | Xanthomonadaceae | *Stenotrophomonas* | *NA* |  |
| **Pieridae** | | | | | | | | |
| ***Pieris rapae*** | L | Artificial diet. | CB, CS | Actinobacteria | Corynebacteriaceae | *Corynebacterium* | *NA* |  |
|  |  |  |  |  | Propionibacteriaceae | *Propionibacterium* | *NA* |  |
|  |  |  |  | Alphaproteobacteria | Acetobacteraceae | *Asaia* | *NA* |  |
|  |  |  |  |  | Methylobacteriaceae | *Methylobacterium* | *NA* |  |
|  |  |  |  |  | Rhizobiaceae | *Rhizobium* | *NA* |  |
|  |  |  |  |  | Acetobacteraceae | *Roseomonas* | *NA* |  |
|  |  |  |  | Bacteroidetes | Flavobacteriaceae | *Flavobacterium* | *NA* |  |
|  |  |  |  |  |  | *Hymenobacter* | *NA* |  |
|  |  |  |  | Betaproteobacteria | Comamonadaceae | *Acidovorax* | *NA* |  |
|  |  |  |  |  |  | *Comamonas* | *NA* |  |
|  |  |  |  |  | NA | *Imtechium* | *NA* |  |
|  |  |  |  |  | Ralstoniaceae | *Ralstonia* | *NA* |  |
|  |  |  |  | Firmicutes | Bacillaceae | *Bacillus* | *NA* |  |
|  |  |  |  |  | Enterococcaceae | *Enterococcus* | *NA* |  |
|  |  |  |  |  | Lactobacillaceae | *Lactobacillus* | *NA* |  |
|  |  |  |  |  | Staphylococcaceae | *Staphylococcus* | *NA* |  |
|  |  |  |  | Gammaproteobacteria | Moraxellaceae | *Acinetobacter* | *NA* |  |
|  |  |  |  |  | Enterobacteriaceae | *Enterobacter* | *NA* |  |
|  |  |  |  |  |  | *Escherichia* | *NA* |  |
|  |  |  |  |  |  | *Pantoea* | *NA* |  |
|  |  |  |  |  | Moraxellaceae | *Moraxella* | *NA* |  |
|  |  |  |  |  | NA | *Nevskia* | *NA* |  |
|  |  |  |  |  | Pseudomonadaceae | *Pseudomonas* | *NA* | (Robinson et al., 2010) |
| **Plutellidae** | | | | | | | | |
| ***Plutella xyllostella*** | E, L, P | Vegetable field, Artificial diet *Brassica* sp. | CB, CS, HS | Actinobacteria | Microbacteriaceae | *Microbacterium* | *NA* |  |
|  |  |  |  | Alphaproteobacteria | Acetobacteraceae | *NA* | *NA* |  |
|  |  |  |  |  | Bradhyrhizobiaceae | *NA* | *NA* |  |
|  |  |  |  |  | Caulobacteraceae | *NA* | *NA* |  |
|  |  |  |  |  | Hyphomicrobiaceae | *NA* | *NA* |  |
|  |  |  |  |  | Methylobacteriaceae | *NA* | *NA* |  |
|  |  |  |  |  | Rhizobiacae | *NA* | *NA* |  |
|  |  |  |  |  | Rhodospirillaceae | *NA* | *NA* |  |
|  |  |  |  |  | Sphingomonadaceae | *NA* | *NA* |  |
|  |  |  |  | Betaproteobacteria | Alcaligenaceae | *NA* | *NA* |  |
|  |  |  |  |  | Burkholderiaceae | *Burkholderia* | *NA* |  |
|  |  |  |  |  |  | *NA* | *NA* |  |
|  |  |  |  |  | Comamonadaceae | *NA* | *NA* |  |
|  |  |  |  |  | Oxalobacteraceae | *NA* | *NA* |  |
|  |  |  |  | Deltaproteobacteria | Bdellovibrionaceae | *NA* | *NA* |  |
|  |  |  |  |  | Polyangiaceae | *NA* | *NA* |  |
|  |  |  |  | Firmicutes | Bacillaceae | *Bacillus* | *NA* |  |
|  |  |  |  |  | Carnobacteriaceae | *Carnobacterium* | *NA* |  |
|  |  |  |  |  | Enterococcaceae | *Enterococcus* | *NA* |  |
|  |  |  |  |  | Staphylococcaceae | *Staphylococcus* | *NA* |  |
|  |  |  |  | Gammaproteobacteria | Enterobacteriaceae | *Enterobacter* | *Enterobacter aerogenes* |  |
|  |  |  |  |  |  | *Providencia* | *NA* |  |
|  |  |  |  |  |  | *Serratia* | *Serratia marcescens* |  |
|  |  |  |  |  | Pseudomonadaceae | *Pseudomonas* | *NA* |  |
|  |  |  |  |  | Aeromonadales | *NA* | *NA* |  |
|  |  |  |  |  | Enterobacteriaceae | *NA* | *NA* |  |
|  |  |  |  |  | Moraxellaceae | *NA* | *NA* |  |
|  |  |  |  |  | Pseudomonadaceae | *NA* | *NA* |  |
|  |  |  |  |  | Vibrionaceae | *NA* | *NA* |  |
|  |  |  |  |  | Xanthomonadaceae | *NA* | *NA* |  |
|  |  |  |  | Actinobacteria | Micrococcaceae | *Arthrobacter* | *NA* |  |
|  |  |  |  |  | Dermabacteraceae | *Brachybacterium* | *NA* |  |
|  |  |  |  |  | Nocardiaceae | *Rhodococcus* | *Rhodococcus erythropolis* |  |
|  |  |  |  | Alphaproteobacteria | Caulobacteraceae | *Brevundimonas* | *Brevundimonas olei* |  |
|  |  |  |  |  | Brucellaceae | *Ochrobactrum* | *NA* |  |
|  |  |  |  | Betaproteobacteria | Alcaligenaceae | *Alcaligenes* | *Alcaligenes faecalis* |  |
|  |  |  |  | Firmicutes | Bacillaceae | *Bacillus* | *Bacillus cereus* |  |
|  |  |  |  |  |  |  | *Bacillus thuringiensis* |  |
|  |  |  |  |  |  |  | *Bacillus pumilus* |  |
|  |  |  |  |  |  |  | *Bacillus nealsonii* |  |
|  |  |  |  |  |  | *Lysinibacillus* | *NA* |  |
|  |  |  |  |  | Staphylococcaceae | *Staphylococcus* | *Staphylococcus xylosus* |  |
|  |  |  |  | Gammaproteobacteria | Enterobacteriaceae | *Enterobacter* | *NA* |  |
|  |  |  |  |  |  | *Erwinia* | *Erwinia chrysanthemi* |  |
|  |  |  |  |  |  | *Leclercia* | *Leclercia adecarboxylata* |  |
|  |  |  |  |  |  |  | *NA* | (Xia et al., 2013; Lin et al., 2014; Lin et al., 2015) |
|  |  |  |  |  |  | *Serratia* | *NA* |  |
|  |  |  |  |  | Pseudomonadaceae | *Pseudomonas* | *Pseudomonas cedrina* |  |
|  |  |  |  |  | Xanthomonadaceae | *Stenotrophomonas* | *Stenotrophomonas maltophilia* |  |
| **Sphingidae** | | | | | | | | |
| ***Hyles euphorbiae*** | L | *Euphorbia sp.* | HS | Actinobacteria | Propionibacterineae | *Nocardiodes* | *NA* |  |
|  |  |  |  |  | Streptomycetaceae | *Streptomyces* | *NA* |  |
|  |  |  |  | Alphaproteobacteria | Rhizobiaceae | *Agrobacterium* | *NA* |  |
|  |  |  |  |  | Sphingomonadaceae | *Sphingomonas* | *NA* |  |
|  |  |  |  | Betaproteobacteria | Burkholderiaceae | *NA* | *NA* |  |
|  |  |  |  | Firmicutes | Enterococcaceae | *Enterococcus* | *NA* |  |
|  |  |  |  |  |  |  | *Enterococcus casseliflavus* |  |
|  |  |  |  |  |  | *NA* | *NA* |  |
|  |  |  |  |  | Bacillaceae | *NA* | *NA* |  |
|  |  |  |  |  | Staphylococcaceae | *Staphylococcus* | *NA* |  |
|  |  |  |  | Gammaproteobacteria | Enterobacteriaceae | *Citrobacter* | *NA* |  |
|  |  |  |  |  |  | *Erwinia* | *NA* |  |
|  |  |  |  |  |  | *NA* | *NA* |  |
|  |  |  |  |  | Xantomonadaceae | *NA* | *NA* |  |
|  |  |  |  |  | Pseudomonadaceae | *Pseudomonas* | *NA* | (Vilanova et al., 2016) |
| ***Manduca sexta*** | E, L | Artificial diet, *Nicotiana tabacum* | CB, HS | Actinobacteria | Corynebacteriaceae | *Corynebacterium* | *NA* |  |
|  |  |  |  |  | Microbacteriaceae | *Curtobacterium* | *NA* |  |
|  |  |  |  |  |  | *Microbacterium* | *NA* |  |
|  |  |  |  |  | Micrococcaceae | *Kocuria* | *NA* |  |
|  |  |  |  |  |  | *Micrococcus* | *NA* |  |
|  |  |  |  | Alphaproteobacteria | Sphingomonadaceae | *Sphingomonas* | *NA* |  |
|  |  |  |  | Bacteroidetes | Flavobacteriaceae | *Flavobacterium* | *Flavobacterium hydatis* |  |
|  |  |  |  |  |  |  | *NA* |  |
|  |  |  |  | Betaproteobacteria | Burkholderiaceae | *Burkholderia* | *NA* |  |
|  |  |  |  |  |  | *Cupriavidus* | *NA* |  |
|  |  |  |  |  | Comamonadaceae | *Delftia* | *Delftia acidovorans* |  |
|  |  |  |  |  | Ralstoniaceae | *Ralstonia* | *NA* |  |
|  |  |  |  | Firmicutes | Bacillaceae | *Bacillus* | *NA* |  |
|  |  |  |  |  |  |  | *Bacillus licheniformis* |  |
|  |  |  |  |  | Enterococcaceae | *Enterococcus* | *Enterococcus gallinarum* |  |
|  |  |  |  |  |  |  | *Enterococcus casseliflavus* |  |
|  |  |  |  |  |  |  | *Enterococcus saccharolyticus* |  |
|  |  |  |  |  | Lactobacillaceae | *Pediococcus* | *NA* |  |
|  |  |  |  |  | Staphylococcaceae | *Staphylococcus* | *NA* |  |
|  |  |  |  | Gammaproteobacteria | Enterobacteriaceae | *Citrobacter* | *Citrobacter sedlakii* |  |
|  |  |  |  |  |  | *Enterobacter* | *Enterobacter aphidicola* |  |
|  |  |  |  |  |  |  | *Enterobacter cloacae* | (Van Der Hoeven et al., 2008; Brinkmann et al., 2008) |
|  |  |  |  |  |  |  | *Enterobacter aerogenes* |  |
|  |  |  |  |  | Pseudomonadaceae | *Pseudomonas* | *NA* |  |
| **Tortricidae** | | | | | | | | |
| ***Choristoneura fumiferana*** | L | *Abies balsamea, Picea mariana,* Artificial diet. | HS | Alphaproteobacteria | Bradyrhizobiaceae | *Bradyrhizobium* | *NA* |  |
|  |  |  |  |  | Methylobacteriaceae | *Methylobacterium* | *NA* |  |
|  |  |  |  | Bacteroidetes | Chitinophagaceae | *Hydrotalea* | *Hydrotalea flava* |  |
|  |  |  |  |  |  | *Sediminibacterium* | *Sediminibacterium ginsengisoli* |  |
|  |  |  |  |  |  |  | *Sediminibacterium ginsengisoli* |  |
|  |  |  |  | Betaproteobacteria | Oxalobacteraceae | *Janthinobacterium* | *NA* |  |
|  |  |  |  | Gammaproteobacteria | Enterobacteriaceae | *Eschlerichia* | *NA* |  |
|  |  |  |  |  | Pseudomonadaceae | *Pseudomonas* | *NA* |  |
|  |  |  |  |  |  |  | *NA* |  |
|  |  |  |  |  |  |  | *NA* |  |
|  |  |  |  |  |  | *Rugamonas* | *Rugamonas rubra* |  |
|  |  |  |  |  |  |  | *Rugamonas rubra* |  |
|  |  |  |  |  |  |  | *Rugamonas rubra* | (Landry et al., 2015) |

REFERENCES

Anand, A.A.P., Vennison, S.J., Sankar, S.G., Prabhu, D.I.G., Vasan, P.T., Raghuraman, T., et al. (2010). Isolation and characterization of bacteria from the gut of *Bombyx mori* that degrade cellulose, xylan, pectin and starch and their impact on digestion. *Journal of Insect Science* 10(1)**,** 107. doi: Artn 107 Doi 10.1673/031.010.10701.

Brinkmann, N., Martens, R., and Tebbe, C.C. (2008). Origin and diversity of metabolically active gut bacteria from laboratory-bred larvae of *Manduca sexta* (Sphingidae, Lepidoptera, Insecta). *Appl Environ Microbiol* 74(23)**,** 7189-7196. doi: 10.1128/AEM.01464-08.

Broderick, N.A., Raffa, K.F., Goodman, R.M., and Handelsman, J. (2004). Census of the bacterial community of the gypsy moth larval midgut by using culturing and culture-independent methods. *Applied and Environmental Microbiology* 70(1)**,** 293-300. doi: 10.1128/Aem.70.1.293-300.2004.

Chen, B., Teh, B.S., Sun, C., Hu, S., Lu, X., Boland, W., et al. (2016). Biodiversity and Activity of the Gut Microbiota across the Life History of the Insect Herbivore *Spodoptera littoralis*. *Sci Rep* 6**,** 29505. doi: 10.1038/srep29505.

Dantur, K.I., Enrique, R., Welin, B., and Castagnaro, A.P. (2015). Isolation of cellulolytic bacteria from the intestine of *Diatraea saccharalis* larvae and evaluation of their capacity to degrade sugarcane biomass. *Amb Express* 5(1)**,** 15. doi: ARTN 15 10.1186/s13568-015-0101-z.

Eski, A., Cakici, F.O., Gullu, M., Muratoglu, H., Demirbag, Z., and Demir, I. (2015). Identification and pathogenicity of bacteria in the Mediterranean corn borer *Sesamia nonagrioides* Lefebvre (Lepidoptera: Noctuidae). *Turkish Journal of Biology* 39(1)**,** 31-48. doi: 10.3906/biy-1402-69.

Haloi, K., Kalita, M.K., Nath, R., and Devi, D. (2016). Characterization and pathogenicity assessment of gut-associated microbes of muga silkworm *Antheraea assamensis* Helfer (Lepidoptera: Saturniidae). *J Invertebr Pathol* 138**,** 73-85. doi: 10.1016/j.jip.2016.06.006.

Hammer, T.J., McMillan, W.O., and Fierer, N. (2014). Metamorphosis of a butterfly-associated bacterial community. *PLoS One* 9(1)**,** e86995. doi: 10.1371/journal.pone.0086995.

He, C., Nan, X., Zhang, Z., and Li, M. (2013). Composition and diversity analysis of the gut bacterial community of the Oriental armyworm, *Mythimna separata*, determined by culture-independent and culture-dependent techniques. *J Insect Sci* 13(165)**,** 165. doi: 10.1673/031.013.16501.

Hernandez-Flores, L., Llanderal-Cazares, C., Guzman-Franco, A.W., and Aranda-Ocampo, S. (2015). Bacteria Present in *Comadia redtenbacheri* Larvae (Lepidoptera: Cossidae). *J Med Entomol* 52(5)**,** 1150-1158. doi: 10.1093/jme/tjv099.

Landry, M., Comeau, A.M., Derome, N., Cusson, M., and Levesque, R.C. (2015). Composition of the Spruce Budworm (*Choristoneura fumiferana*) Midgut Microbiota as Affected by Rearing Conditions. *Plos One* 10(12)**,** e0144077. doi: ARTN e0144077 10.1371/journal.pone.0144077.

Lin, X.L., Kang, Z.W., Pan, Q.J., and Liu, T.X. (2015a). Evaluation of five antibiotics on larval gut bacterial diversity of *Plutella xylostella* (Lepidoptera: Plutellidae). *Insect Sci* 22(5)**,** 619-628. doi: 10.1111/1744-7917.12168.

Lin, X.L., Pan, Q.J., Tian, H.G., Douglas, A.E., and Liu, T.X. (2015b). Bacteria abundance and diversity of different life stages of *Plutella xylostella* (Lepidoptera: Plutellidae), revealed by bacteria culture-dependent and PCR-DGGE methods. *Insect Sci* 22(3)**,** 375-385. doi: 10.1111/1744-7917.12079.

Osborn, F., Berlioz, L., Vitelli-Flores, J., Monsalve, W., Dorta, B., and Rodriguez Lemoine, V. (2002). Pathogenic effects of bacteria isolated from larvae of *Hylesia metabus* Crammer (Lepidoptera: Saturniidae). *J Invertebr Pathol* 80(1)**,** 7-12.

Pinto-Tomás, A., Uribe-Lorío, L., Blanco, J., Fontecha, G., Rodríguez, C., Mora, M., et al. (2007). Actividades enzimáticas en aislamientos bacterianos de tractos digestivos de larvas y del contenido de pupas de *Automeris zugana* y *Rothschildia lebeau* (Lepidoptera: Saturniidae). *Revista de biología tropical* 55(2)**,** 401.

Pinto-Tomas, A.A., Sittenfeld, A., Uribe-Lorio, L., Chavarria, F., Mora, M., Janzen, D.H., et al. (2011). Comparison of midgut bacterial diversity in tropical caterpillars (Lepidoptera: Saturniidae) fed on different diets. *Environ Entomol* 40(5)**,** 1111-1122. doi: 10.1603/EN11083.

Robinson, C.J., Schloss, P., Ramos, Y., Raffa, K., and Handelsman, J. (2010). Robustness of the bacterial community in the cabbage white butterfly larval midgut. *Microb Ecol* 59(2)**,** 199-211. doi: 10.1007/s00248-009-9595-8.

Secil, E.S., Sevim, A., Demirbag, Z., and Demir, I. (2012). Isolation, characterization and virulence of bacteria from *Ostrinia nubilalis* (Lepidoptera: Pyralidae). *Biologia* 67(4)**,** 767-776. doi: 10.2478/s11756-012-0070-5.

Sevim, A., Sevim, E., Demirci, M., and Sandalli, C. (2016). The internal bacterial diversity of stored product pests. *Annals of Microbiology* 66(2)**,** 749-764. doi: 10.1007/s13213-015-1155-5.

Sittenfeld, A., Uribe-Lorío, L., Mora, M., Nielsen, V., Arrieta, G., and H Janzen, D. (2002). Does a polyphagous caterpillar have the same gut microbiota when feeding on different species of food plants? *Revista de biología tropical* 50(2)**,** 547-560.

Snyman, M., Gupta, A.K., Bezuidenhout, C.C., Claassens, S., and van den Berg, J. (2016). Gut microbiota of *Busseola fusca* (Lepidoptera: Noctuidae). *World J Microbiol Biotechnol* 32(7)**,** 115. doi: 10.1007/s11274-016-2066-8.

Staudacher, H., Kaltenpoth, M., Breeuwer, J.A., Menken, S.B., Heckel, D.G., and Groot, A.T. (2016). Variability of Bacterial Communities in the Moth *Heliothis virescens* Indicates Transient Association with the Host. *PLoS One* 11(5)**,** e0154514. doi: 10.1371/journal.pone.0154514.

Tang, X., Freitak, D., Vogel, H., Ping, L., Shao, Y., Cordero, E.A., et al. (2012). Complexity and variability of gut commensal microbiota in polyphagous lepidopteran larvae. *PLoS One* 7(7)**,** e36978. doi: 10.1371/journal.pone.0036978.

Thakur, A., Dhammi, P., Saini, H.S., and Kaur, S. (2015). Pathogenicity of bacteria isolated from gut of *Spodoptera litura* (Lepidoptera: Noctuidae) and fitness costs of insect associated with consumption of bacteria. *J Invertebr Pathol* 127**,** 38-46. doi: 10.1016/j.jip.2015.02.007.

van der Hoeven, R., Betrabet, G., and Forst, S. (2008). Characterization of the gut bacterial community in *Manduca sexta* and effect of antibiotics on bacterial diversity and nematode reproduction. *FEMS Microbiol Lett* 286(2)**,** 249-256. doi: 10.1111/j.1574-6968.2008.01277.x.

Vilanova, C., Baixeras, J., Latorre, A., and Porcar, M. (2016). The Generalist Inside the Specialist: Gut Bacterial Communities of Two Insect Species Feeding on Toxic Plants Are Dominated by *Enterococcus* sp. *Front Microbiol* 7**,** 1005. doi: 10.3389/fmicb.2016.01005.

Visotto, L.E., Oliveira, M.G., Ribon, A.O., Mares-Guia, T.R., and Guedes, R.N. (2009). Characterization and identification of proteolytic bacteria from the gut of the velvetbean caterpillar (Lepidoptera: Noctuidae). *Environ Entomol* 38(4)**,** 1078-1085.

Walenciak, O., Zwisler, W., and Gros, E.M. (2002). Influence of Myriophyllum spicatum-derived tannins on gut microbiota of its herbivore *Acentria ephemerella*. *J Chem Ecol* 28(10)**,** 2045-2056.

Xia, X., Zheng, D., Zhong, H., Qin, B., Gurr, G.M., Vasseur, L., et al. (2013). DNA sequencing reveals the midgut microbiota of diamondback moth, *Plutella xylostella* (L.) and a possible relationship with insecticide resistance. *PLoS One* 8(7)**,** e68852. doi: 10.1371/journal.pone.0068852.

Xiang, H., Wei, G.F., Jia, S., Huang, J., Miao, X.X., Zhou, Z., et al. (2006). Microbial communities in the larval midgut of laboratory and field populations of cotton bollworm (*Helicoverpa armigera*). *Can J Microbiol* 52(11)**,** 1085-1092. doi: 10.1139/w06-064.

Yu, H.W., Wang, Z.K., Liu, L., Xia, Y.X., Cao, Y.Q., and Yin, Y.P. (2008). Analysis of the intestinal microflora in *Hepialus gonggaensis* larvae using 16S rRNA sequences. *Current Microbiology* 56(4)**,** 391-396. doi: 10.1007/s00284-007-9078-4.

Zaspel, J.M., and Hoy, M.A. (2008). Microbial Diversity Associated with the Fruit-Piercing and Blood-Feeding Moth *Calyptra thalictri* (Lepidoptera: Noctuidae). *Annals of the Entomological Society of America* 101(6)**,** 1050-1055. doi: Doi 10.1603/0013-8746-101.6.1050.
